# Cannabidiol potentiates p53-driven autophagic cell death in non-small cell lung cancer following DNA damage: a novel synergistic approach beyond canonical pathways

**DOI:** 10.1038/s12276-025-01444-x

**Published:** 2025-05-01

**Authors:** Youngsic Jeon, Taejung Kim, Hyukjoon Kwon, Young Nyun Park, Tae-Hyung Kwon, Min Hong, Kyung-Chul Choi, Jungyeob Ham, Young-Joo Kim

**Affiliations:** 1 Institute of Natural Products, Korea Institute of Science and Technology, Gangneung, Republic of Korea; 2 Natural Product Applied Science, KIST School, University of Science and Technology, Gangneung, Republic of Korea; 3 Department of Pathology, Graduate School of Medical Science, Brain Korea 21 Project, Yonsei University College of Medicine, Seoul, Republic of Korea; 4 Institute of Biological Resources, Chuncheon Bioindustry Foundation, Chuncheon, Republic of Korea; 5 Department of Biochemistry and Molecular Biology, Brain Korea 21 project, Asan Medical Center, University of Ulsan College of Medicine, Seoul, Republic of Korea; 6 NeoCannBio Co. Ltd, Seoul, Republic of Korea

**Keywords:** Chemotherapy, Apoptosis

## Abstract

The search for more effective and safer cancer therapies has led to an increasing interest in combination treatments that use well-established agents. Here we explore the potential of cannabidiol (CBD), a compound derived from cannabis, to enhance the anticancer effects of etoposide in non-small cell lung cancer (NSCLC). Although CBD is primarily used to manage childhood epilepsy, its broader therapeutic applications are being actively investigated, particularly in oncology. Our results revealed that, among various tested chemotherapeutic drugs, etoposide showed the most significant reduction in NSCLC cell viability when combined with CBD. To understand this synergistic effect, we conducted extensive transcriptomic and proteomic profiling, which showed that the combination of CBD and etoposide upregulated genes associated with autophagic cell death while downregulating key oncogenes known to drive tumor progression. This dual effect on cell death and oncogene suppression was mediated by inactivation of the PI3K–AKT–mTOR signaling pathway, a crucial regulator of cell growth and survival, and was found to be dependent on the p53 status. Interestingly, our analysis revealed that this combination therapy did not rely on traditional cannabinoid receptors or transient receptor potential cation channels, indicating that CBD exerts its anticancer effects through novel, noncanonical mechanisms. The findings suggest that the combination of CBD with etoposide could represent a groundbreaking approach to NSCLC treatment, particularly in cases where conventional therapies fail. By inducing autophagic cell death and inhibiting oncogenic pathways, this therapeutic strategy offers a promising new avenue for enhancing treatment efficacy in NSCLC, especially in tumors with p53 function.

## Cannabidiol boosts etoposide efficacy in lung cancer treatment

Lung cancer is a major health issue, with non-small cell lung cancer (NSCLC) being the most common type. Researchers explored combining cannabidiol (CBD), a compound from cannabis, with etoposide, a chemotherapy drug, to improve treatment for NSCLC.

The study involved laboratory experiments on NSCLC cells and mice to test the effects of CBD and etoposide together. They used various methods including RNA sequencing (a technique to study gene activity) and protein analysis to understand how the combination works. The researchers found that CBD enhances the effect of etoposide, leading to increased cancer cell death through a process called autophagy (a type of cell self-destruction). The results suggest that this combination could be more effective than current treatments alone. The study highlights the potential of using CBD to boost chemotherapy effectiveness in NSCLC.

This summary was initially drafted using artificial intelligence, then revised and fact-checked by the author.

## Introduction

Lung cancer is typically categorized into non-small cell lung cancer (NSCLC) and small cell lung cancer (SCLC). NSCLC, accounting for 85% lung cancers, is characterized by its heterogeneity and slower growth than that of SCLC. Despite advances in treatment, NSCLC often leads to poor prognosis due to its aggressiveness^1,2^. Current standard chemotherapeutic regiments for NSCLC include cisplatin, carboplatin and paclitaxel. However, these treatments are often limited by low response rate, the emergence of drug resistance and significant adverse effects. This underscores the urgent need for more effective and safer therapeutic strategies.

Etoposide, a well-established chemotherapeutic agent used to treat SCLC and various other malignancies owing to its broad-spectrum efficacy, inhibits topoisomerase II activity, leading to DNA damage and cytotoxicity. However, its application in NSCLC has been limited, primarily because of its significantly low efficacy in this subtype. This is probably due to the distinct biological characteristics and chemotherapy response profiles between SCLC and NSCLC, necessitating the exploration of novel combination therapies to enhance treatment outcomes in NSLCL^3,4^.

Cannabidiol (CBD) has been advocated for various health conditions. Its therapeutic efficiency against severe childhood epilepsy progressions, such as Dravet and Lennox–Gastaut syndromes, which are usually resistant to conventional antiseizure treatments, is substantially supported by scientific evidence. Previous reports have shown that CBD could decrease seizure frequency and, sometimes, cease them completely. With CBD as a primary ingredient, Epidiolex was the first cannabis-derived drug approved by the Food and Drug Administration to treat these conditions^5,6^. However, the effects of CBD on NSCLC and its potential in combination therapy remain unknown.

In NSCLC, combination treatment using chemotherapeutic agents, including cisplatin, carboplatin and paclitaxel, has shown promising results. For instance, combination treatment using paclitaxel and carboplatin improved survival rates and reduced toxicity compared with single-agent therapies^7,8^. Conversely, combining cisplatin with paclitaxel has been shown to significantly increase response rates, although some associated toxicities have been reported^8^. Collectively, combination therapies that utilize well-established and safe chemotherapeutic agents offer a promising approach to improving treatment efficacy and safety.

This study aims to investigate the synergetic effect of CBD when combined with etoposide in NSCLC cells. Notably, we performed transcriptomic and proteomic profiling to elucidate the key molecular pathways using RNA sequencing (RNA-seq) and multiphospho-kinase arrays. We hypothesized that the combination treatment using CBD and etoposide could effectively influence autophagic cell death, presenting a potential breakthrough in the management of NSCLC.

## Materials and methods

### CBD information

CBD and its derivatives, including tetrahydrocannabinol (THC), tetrahydrocannabinolic acid (THCA) and cannabidiolic acid (CBDA), were obtained on the basis of previous studies^9^. The Ministry of Food and Drug Safety and the Seoul Regional Food and Drug Administration approved the allocation and transfer of cannabis for research purposes (approval numbers 1564 and 1979).

### Cell culture

We procured the NSCLC cell line A549 from the American Type Culture Collection. The NCI-H1703, NCI-H358, NCI-H1299, Calu-3 and HEL299 cell lines were purchased from the Korean Cell Line Bank. All cell lines were routinely maintained in RPMI1640 medium (Gibco), supplemented with 10% fetal bovine serum (Gibco), 100 U/ml penicillin and 100 μg/ml streptomycin, at 37 °C in a humidified atmosphere with 5% CO_2_.

### Assessment of cell viability using the WST-8 assay

Cells (1 × 10^4^ per well) were incubated into 96-well plates for 24 h. Subsequently, the cells were maintained with various concentrations of etoposide and/or CBD for 24 h and 48 h. We used the WST-8 assay kit (Biomax) to evaluate cell viability following the manufacturer’s guidelines.

### Western blotting

Protein expression and phosphorylation levels were analyzed via western blotting as described previously^9^. Cells were lysed in RIPA buffer (Cell Signaling Technology) supplemented with protease inhibitor cocktail and phosphatase inhibitor cocktail (Cell Signaling Technology) on ice for 30 min. The lysates were clarified by centrifugation at 14,000 rpm and 4 °C for 15 min, and the supernatants were collected. Proteins (30–50 μg per lane) were separated by electrophoresis on NuPAGE 4–12% Bis–Tris gels (Invitrogen), transferred onto PVDF membranes and probed with specific primary and secondary antibodies. Signal detection was performed using SuperSignal West Femto (Thermo Fisher Scientific) and visualized using a LAS 4000 system (Fujifilm). Details of the applied antibodies and the experimental conditions are provided in Supplementary Table 1.

### Multiphospho-kinase assay

To profile the phospho-kinase activity, the Human Phospho-Kinase Array Kit (R&D Systems) was used. Whole-cell extracts (200 μg per A and B membranes) were prepared and analyzed following the manufacturer’s protocol. Protein phosphorylation was visualized using the LAS 4000 system (Fujifilm).

### Flow cytometry analysis

To detect the apoptotic cells, A549 cells were divided into control, CBD (15 μM), etoposide (20 μM) and combined treatment groups. After 48 h of treatment, we evaluated the cells using flow cytometry with the FITC Annexin V apoptosis detection kit (BD Biosciences) as described previously^9^, and the data were processed using FlowJo software (TreeStar). At least 1000 cells were analyzed for each sample.

### In vivo experiments

We subcutaneously injected A549 cells (1 × 10^7^ cells) suspended in RPMI (100 µl) mixtures into the right rear dorsal flank of BALB/c-nude mice (4 weeks old, weighing 18 ± 2 g, Orient Bio). When the tumor volume reached approximately 50 mm^2^, the mice were randomly divided into five groups: control, CBD (10 mg/kg), etoposide (5 mg/kg, Sigma-Aldrich), low-dose etoposide (1 mg/kg) + CBD, and high-dose etoposide (5 mg/kg) + CBD. CBD and etoposide were dispersed in 0.5% carboxymethylcellulose and given orally once daily at a concentration ranging from 1 to 10 mg/kg body weight. During the first 4 weeks, we weighed the mice and recorded any uncommon behavior and death. Finally, the animals were anesthetized using ether and euthanized. All the animal experimental procedures were approved and followed the Institutional Animal Care and Use Committees of KIST guidelines (approval no. KIST-IACUC-2023-046).

### RNA-seq data processing and transcriptome profiling

For transcriptome analysis, A549 cells were plated into six-well plates at 3 × 10^5^ cells per well and incubated for 24 h before being treated with CBD (15 μM), etoposide (20 μM) and merge. After 24 h of treatment, total RNA was extracted using the RNeasy kit (Qiagen). RNA-seq profiling was performed using Illumina Hiseq4000. To compute the fragments per kilobase of transcript per million mapped reads (FPKM), we applied Tophat and Cufflinks^10^. Using the obtained FPKM values, transcriptome profiling was performed in the R environment (https://www.r-project.org/). We applied transcriptome profiling and gene set enrichment analysis (GSEA), as previously described^11^. We analyzed the gene sets using STRING (https://string-db.org/) based on Kyoto Encyclopedia of Genes and Genomes (KEGG) and UniProt databases. Also, we calculated GSEA using KEGG (http://www.genome.jp/kegg/) and REAC (reactome, http://www.reactome.org/) databases. The list of oncogenes (*n* = 674) and tumor suppressor genes (*n* = 1,088) was retrieved from previous reports^12,13^. The details of the data and references are summarized in Supplementary Table 2.

### Immunofluorescence

For immunofluorescence, we followed preestablished methods. A549 cells were plated in coverglasses (16 mm) at a density of 1 × 10^4^ cells per well and incubated for 24 h before treatment with CBD (15 μM), etoposide (20 μM) and merge. A549 cells were fixed, permeabilized and blocked with 2% bovine serum albumin for 1 h. Then, the cells were stained with anti-LC3B and anti-SQSTM1/p62 antibody in 2% bovine serum albumin for 24 h at 4 °C, followed by staining with Alexa Flour 488-conjugated goat anti-rabbit IgG as the secondary antibody for 1 h at 37 °C. Finally, we washed and mounted the cells with DAKO Fluorescent Mounting Medium (DakoCytomation). The stained cells were evaluated using an EVOS M5000 imaging system (Invitrogen). The antibodies and the experiment conditions are listed in Supplementary Table 1.

### Monodansylcadaverine (MDC) assay

The cells (2 × 10^6^) were seeded into a 100-mm dish and treated with etoposide and/or CBD for 12 h. Subsequently, whole-cell extracts were prepared and assessed using a human phospho-kinase array system (R&D Systems) to detect phospho-kinase activity following the manufacturer’s guidelines.

### Transient and stable cell lines

The tagged cannabinoid receptors (*CNR1* and *CNR2*) and *TP53* genes were cloned into the pCDH-CMV-EF1-puro vector after amplifying their respective genes using total RNA extracted from HEK293 and A549 cells, respectively. We conducted PCR using precise primers containing XbaI (NEB) and NotI (NEB) restriction sites with CloneAmp HiFi PCR Premix (Thermo Fisher Scientific). Next, we cloned the PCR products into the linearized vector using the In-Fusion cloning system (Takara Bio). Primer sequences and thermal cycling details are provided in Supplementary Table 3.

A549, NCI-H358 and NCI-H1299 cells were transfected with pCDH-CMV-EF1-puro containing tagged *CNRs* and *TP53* coding sequences, along with the gag-pol and VSV-G plasmids, using Lipofectamine 3000 (Invitrogen). Stable cell lines expressing *CNRs* and *TP53* were established by selecting transfected cells with 0.5–1.0 µg/ml puromycin (Sigma-Aldrich) for 4 weeks.

*TP53*, *TRPV1* and *TRPV2* siRNA (MISSION select predesigned siRNAs, Sigma-Aldrich) and MISSION siRNA Universal Negative Control (Sigma-Aldrich) were transfected into A549 cells using Lipofectamine RNAiMAX Transfection Reagent (Invitrogen) according to the manufacturer’s recommendations.

### Statistical analysis

The results are presented as mean ± s.d. from at least three independent experiments. Statistical analysis was carried out using Student’s *t*-tests and one-way ANOVA, with significance defined at *P* < 0.05.

## Results

### Low concentrations of CBD did not induce cell death in A539 cells

CBD has been shown to exhibit anticancer activity and influence apoptotic cell death at high doses (above 40 μM)^9,14^. This study aimed to investigate the potential of CBD to improve the therapeutic effects of conventional anticancer treatments. Therefore, we evaluated the optimal concentration of CBD using A549 cells to assess its synergistic effects. CBD exhibited obvious cytotoxicity (Supplementary Fig. 1a) and induced apoptosis by altering the expression patterns of related markers such as poly(ADP-ribose) polymerase (PARP), caspases 8 and 9, and Bcl2-associated X (Bax) at concentrations above 25 μM (Supplementary Fig. 1b). Therefore, a concentration of 15 μM CBD was selected for subsequent experiments in A549 cells because it was noncytotoxic.

### CBD increases sensitivity to etoposide in A549 cells

**Fig. 1:**
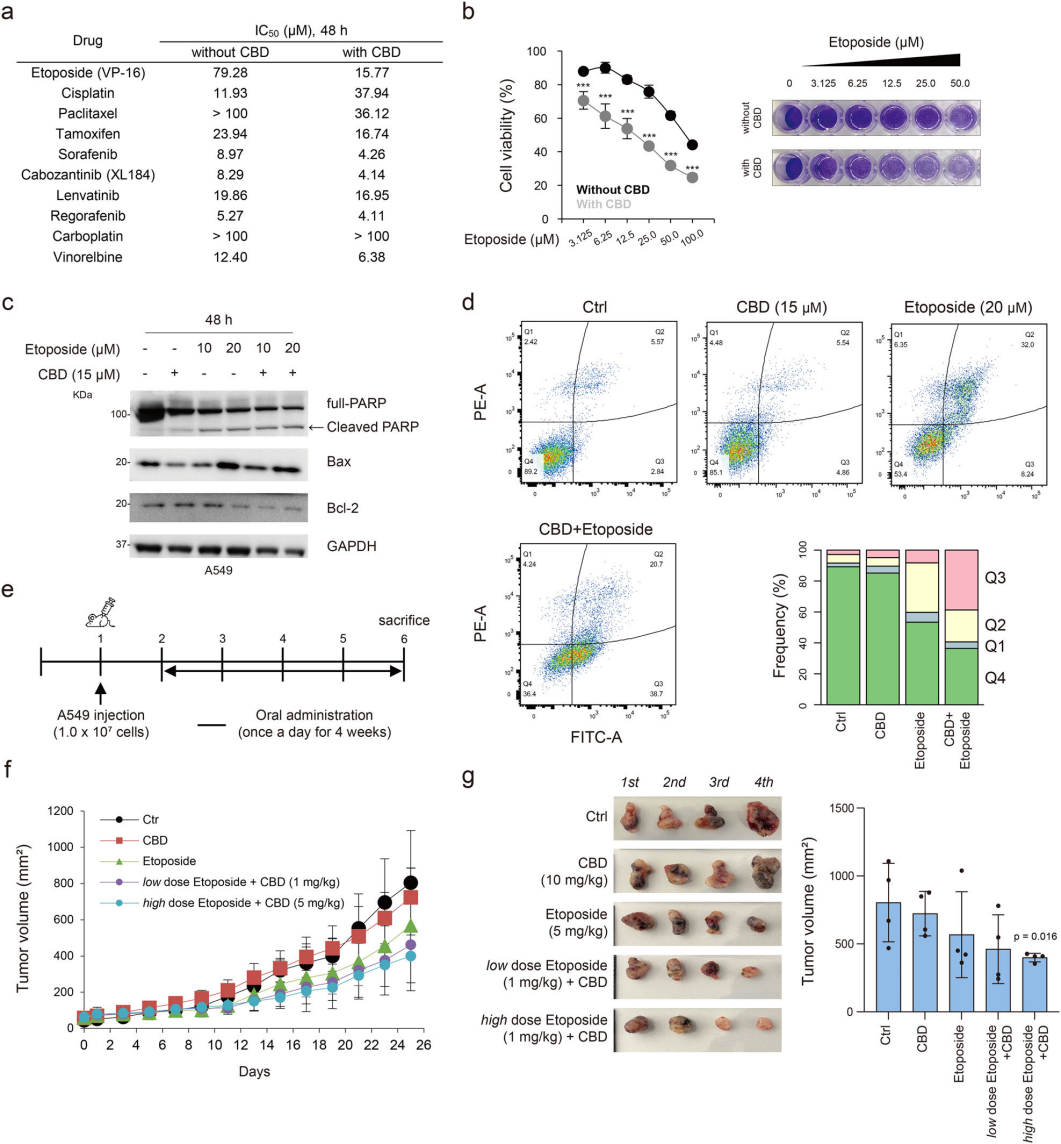
CBD sensitivity to etoposide in A549 cells. **a** IC_50_ values of anticancer drugs with and without CBD. **b** Cell viability (left) and crystal violet staining (right) of A549 cells treated with etoposide, with or without CBD, for 48 h. Statistical significance is indicated (***P < 0.001; Student’s *t*-test). **c** Western blot analysis of cleaved PARP, Bax and Bcl-2, normalized to GAPDH. **d** Top: flow cytometry analysis of A549 cells under the indicated conditions where early apoptotic cells are indicated by the increased fluorescence intensity of FITC-conjugated Annexin V. Bottom: a bar plot showing the frequency of cell population. **e** A diagram of the experimental schedule for the A549 xenograft mouse model. **f** A point plot showing the tumor size on the indicated dates across the groups (control (Ctrl), CBD, etoposide (5 mg/kg), low-dose etoposide (1 mg/kg) + CBD, and high-dose etoposide (5 mg/kg) + CBD). **g** Representative images of dissected tumors from each experimental group. Results are presented as mean ± s.d. (*n* = 4 per group). Statistical significance is indicated (versus Ctrl, ***P < 0.001; Student’s *t*-test).

CBD has been shown to act synergistically with anticancer drugs to elicit cell death^14^. Therefore, we evaluated the viability of A549 cells treated with CBD in combination with well-known anticancer drugs (including etoposide (VP-16), cisplatin, paclitaxel and tamoxifen). Among these combinations, etoposide with CBD exhibited the highest inhibitory effect, as demonstrated by the suppression of cell proliferation (half maximal inhibitory concentration; IC_50_ = 15.77 μM; Fig. 1a, b). By contrast, the combination treatment showed no notable inhibitory effect on the normal lung fibroblast cell line, HEL299 (IC_50_ >100 μM; Supplementary Fig. 2). We also investigated the synergistic effects of etoposide with other cannabinoids that have a similar structure to CBD, including CBDA, CBD, THCA and THC, which did not show improved cancer cell toxicity (Supplementary Table 4).

Etoposide is used to treat SCLC, but not NSCLC^1,15,16^. We further explored whether CBD could facilitate the induction of apoptosis by etoposide in A549 cells. Compared with treatment with etoposide alone, combined treatment with CBD resulted in higher levels of apoptotic protein markers, including cleaved PARP and Bax, in a dose-dependent manner. Conversely, the expression of B-cell lymphoma 2 (Bcl-2), an inhibitor of apoptotic stimuli, showed a decreasing pattern (Fig. 1c). We assessed the apoptotic rate using flow cytometry, which showed that the apoptotic cells were significantly higher in the combined treatment than those treated with either CBD or etoposide (*P* < 0.05; Fig. 1d).

Furthermore, we validated our findings using an A549 xenograft model (Fig. 1e). We determined the suitable concentrations of etoposide (5 mg/kg) and CBD (5 and 10 mg/kg) that did not cause substantial body weight loss (Supplementary Fig. 3). The combination treatment significantly reduced the tumor size compared with either treatment alone (Fig. 1f, g). Taken together, our findings suggest that CBD might enhance the sensitivity to etoposide-induced apoptosis in A549 cells.

### Transcriptomic profiling of A549 cells after combination treatment with etoposide

**Fig. 2:**
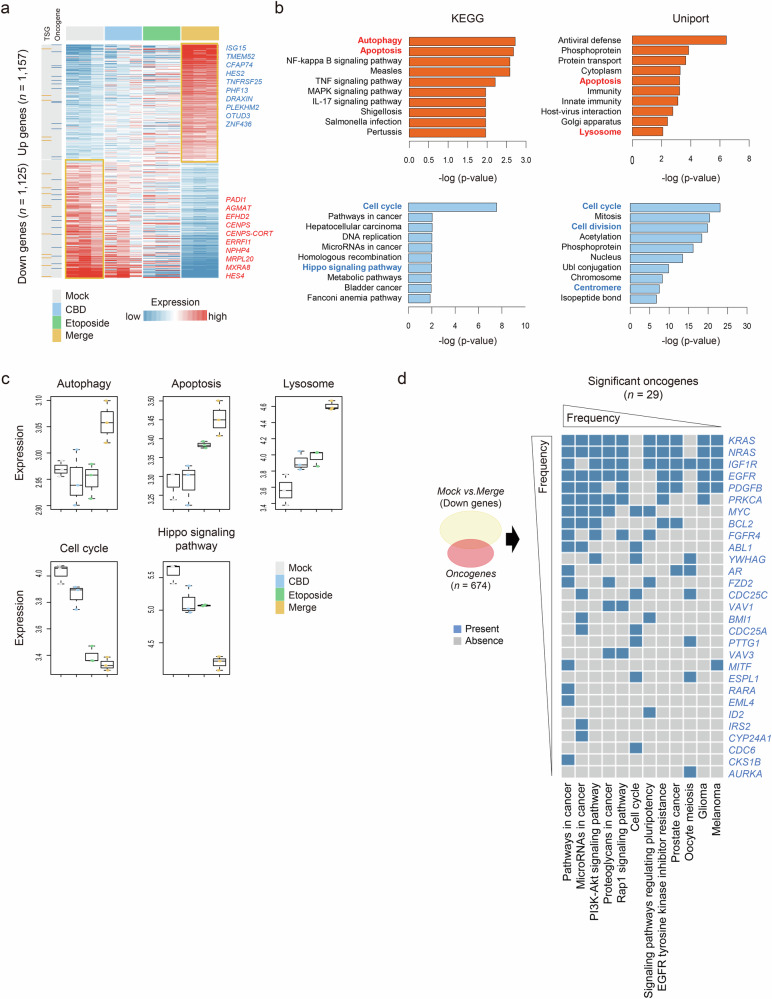
Transcriptomic profiling of combination treatment with etoposide and CBD on A549 cells. **a** A heat map showing the differentially expressed genes (*n* = 2282, permuted *t*-test *P* < 0.05 and fold difference >0.5). The top ten ranked genes, oncogenes and tumor suppressor genes are indicated. **b** Bar plots show differentially enriched gene signatures between mock and merge groups, identified by significant *P* values of gene sets (*P* < 0.05, KEGG and UniProt). **c** Box plots of gene set expression related to autophagy, apoptosis, lysosome, cell cycle and Hippo signaling pathways across groups. **d**, A heat map showing significant differentially oncogenes and their associated signatures across the groups.

We conducted RNA-seq to analyze the transcriptomic profile and explain the fundamental mechanisms underlying the combination treatment using etoposide and CBD on A549 cells (Supplementary Fig. 4a). Unsupervised clustering analysis of the variably expressed genes (median absolute deviation >0.5, *n* = 1,676) demonstrated that the four groups (control, CBD, etoposide and merge (etoposide + CBD)) were clearly classified on the basis of their treatment status (Supplementary Fig. 4b). Furthermore, principal component analysis revealed that the merge groups were notably distributed in separate clusters compared with other groups, reflecting distinct transcriptomic traits (Supplementary Fig. 4c). Next, we identified the robust genes by analyzing the differentially expressed genes between the untreated and combination-treated cells. Two categories were retrieved; ‘Up genes’ (*n* = 1,157) and ‘Down genes’ (*n* = 1,125) (*P* < 0.05, fold change (FC) >1.0; Fig. 2a and Supplementary Table 5). The former genes were significantly associated with autophagy, apoptosis and lysosome signaling pathway-related genes (Fig. 2b, top). Conversely, the later genes were significantly associated with cell cycle and Hippo signaling pathway-related genes (Fig. 2b, bottom). These genes are involved in cancer development^17,18^. We found that the ‘Up genes’ traits reveal the synergistic effect on gene expression patterns (*P* < 0.01; Fig. 2c). In addition, the merge groups showed reduced expression of oncogene-related cancer traits (for example, pathways in cancer, microRNAs in cancer, PI3K–Akt signaling pathway and so on). Among these oncogenes, *KRAS* (FC −0.51), *NRAS* (FC −0.55), *IGF1R* (FC −1.17), *EGFR* (FC −0.95), *PDGFB* (FC −0.92), *PRKCA* (FC −0.59) and *MYC* (FC −1.83), which are highly relevant to the pathogenesis of NSLCL, showed significant differences between the groups (*P* < 0.05; Fig. 2d).

### Combination treatment with CBD and etoposide induces autophagic cell death

**Fig. 3:**
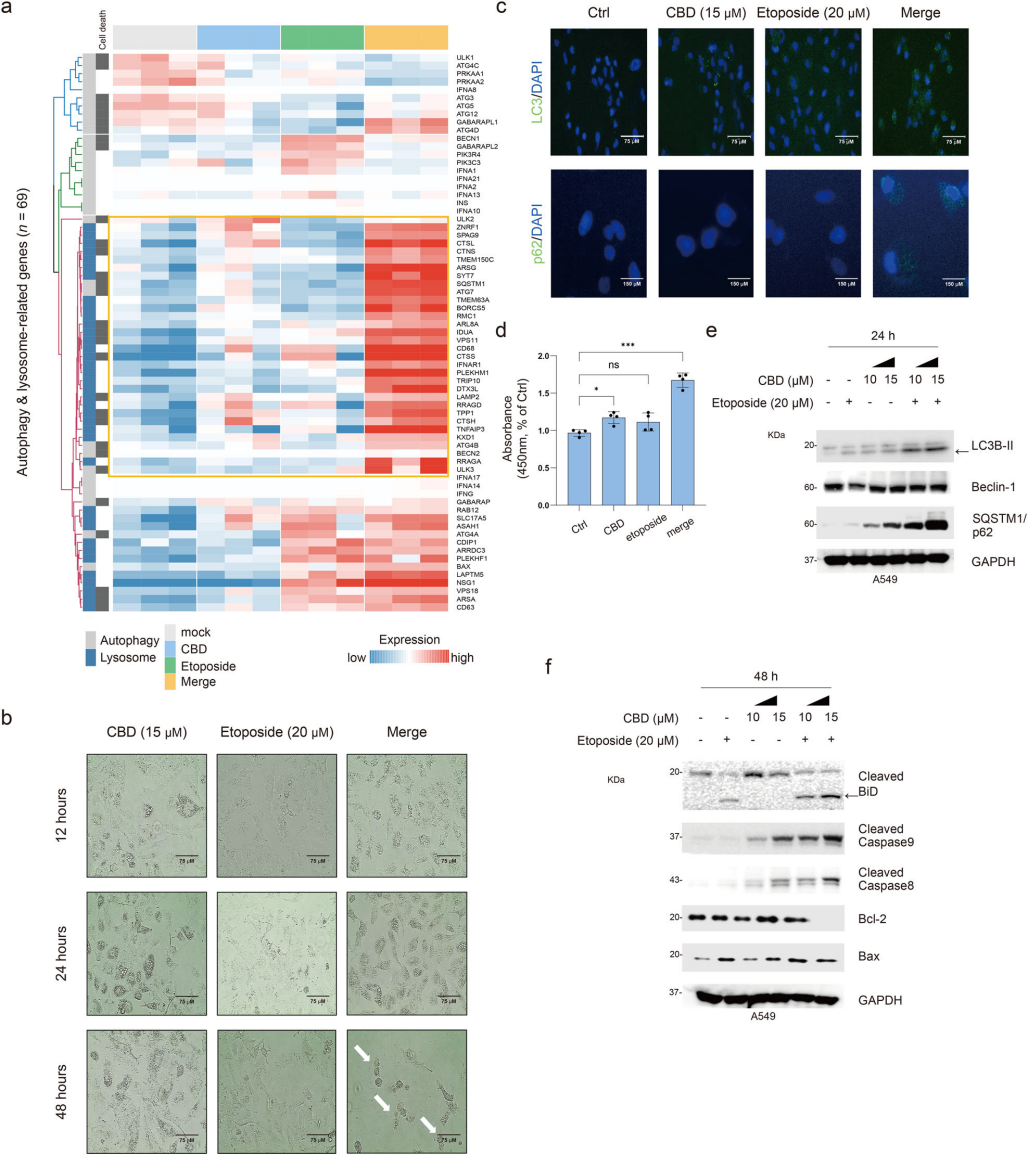
Effect of combination treatment with CBD and etoposide on autophagic cell death. **a** A supervised clustering heat map displaying the autophagy-, lysosome- and cell-death-related genes (*n* = 69) across the groups with each relevant gene indicated. **b** Morphological features of autophagic cell death in A549 cells treated with CBD (15 μM), etoposide (20 μM) and their combination (merge) at 12, 24 and 48 h. White arrows indicate autophagic cell death. **c** Confocal immunofluorescence of LC3B (green), SQSTM1/p62 (green) and DAPI (blue) in A549 cells using the EVOS M5000 imaging system. **d** A bar plot showing the absorbance of MDC, indicating the autophagic traits in A549 cells treated with CBD (15 μM), etoposide (20 μM) and their combination (merge) at 24 h. Statistical significance is indicated (versus Ctrl, *P < 0.05 and ***P < 0.001; Student’s *t*-test). **e** Western blot analysis of LC3B, Beclin and SQSTM1/p62, normalized to GAPDH. **f** Western blot of cleaved BiD, caspases 8 and 9, Bcl-2 and Bax, normalized to GAPDH.

The RNA-seq analysis revealed that autophagy and lysosome traits were notably present in the merge groups. Furthermore, supervised clustering using autophagy and lysosome-related genes (*n* = 69) was significantly upregulated in the merge groups. These genes included cell-death-related genes, such as *SQSTM1* (FC 0.98), *LAMP2* (FC 0.52), *CTSL* (FC 1.32) and *CTNS* (FC 0.65) (Fig. 3a). Morphological analysis further confirmed that the combination treatment promotes autophagy and apoptosis (Fig. 3b). We also observed increased markers of autophagic cell death in the merge groups (Fig. 3c, d). To further validate these findings, we performed a western blot analysis on the A549 cells. The combination treatment elevated LC3B-II and SQSTM1/p62 expression but not Beclin-1, indicating that it induced apoptosis. In addition, this treatment enhanced apoptotic traits, as indicated by increased levels of cleaved BiD, caspases 8 and 9, and Bax, and decreased levels of Bcl-2 (Fig. 3e, f). Consistently, our findings support the hypothesis that CBD promotes etoposide-induced apoptosis through autophagic cell death mechanisms.

**Fig. 4:**
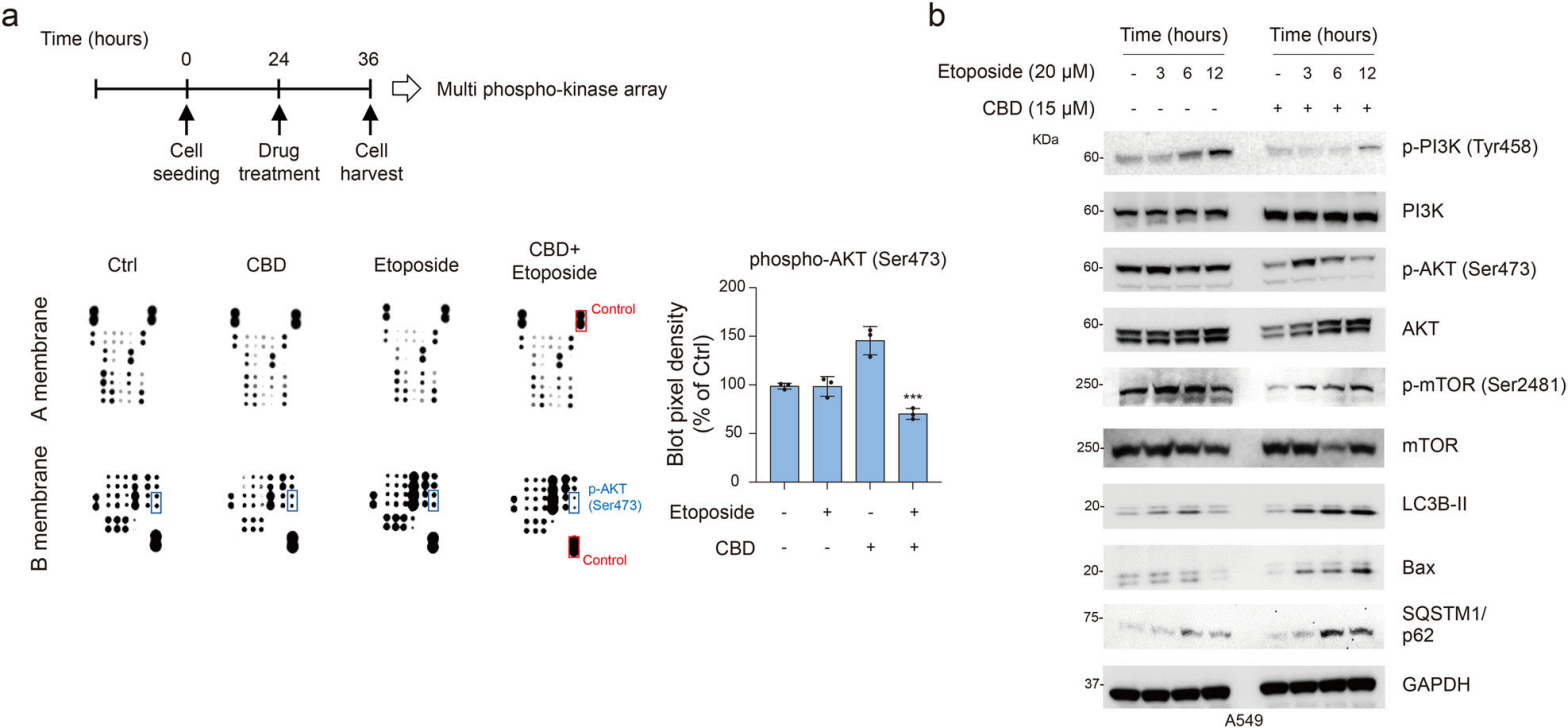
Effect of combination treatment with CBD and etoposide on the PI3K–AKT–mTOR pathway. **a** A diagram of the experimental schedule for A549 cells treated with CBD (15 μM), etoposide (20 μM) and their combination (merge) at 24 h for the multiphospho-kinase array. Left: representative images (membranes A and B) show a selected region of the phospho-kinase array. Right: a box plot shows the pixel density of phospho-AKT. Statistical significance is indicated (versus Ctrl, ***P < 0.001; Student’s *t*-test). **b** Western blot analysis of phospho-PI3K (Tyr458), PI3K, phospho-AKT (Ser473), AKT, phospho-mTOR (Ser2481), mTOR, LC3B, Bax and SQSTM1/p62, normalized to GAPDH.

To explain the fundamental process of the combined treatment of etoposide and CBD, we applied a multiphospho-kinase assay to select the associated signaling pathways. Consequently, we identified putative kinase proteins required for cell multiplication, differentiation, cell cycle regulation and apoptosis. In particular, the levels of phosphorylated serine–threonine kinase protein (AKT) notably decreased after the combination treatment compared with that after a single treatment (Fig. 4a). As the phosphorylation of AKT at Ser473 is linked to phosphoinositide 3-kinase (PI3K) activation, the mammalian target of rapamycin (mTOR) is a central regulatory kinase protein that is crucial for autophagic traits^19,20^. Therefore, we investigated whether the combination treatment between etoposide and CBD led to the inactivation of the PI3K, AKT and mTOR pathways. We observed a marked reduction in the phosphorylation of PI3K, AKT and mTOR and an increase in the expression of autophagic and apoptotic markers (including LC3B-II, Bax and p62) after the combination treatment compared with the single treatment of etoposide (Fig. 4b). These results suggest that the combination treatment could induce autophagic cell death, at least partially, through the inactivation of the PI3K–AKT–mTOR signaling pathway.

### Autophagic cell death induced by combination treatment is associated with the LATS1–p53–PTEN axis

**Fig. 5:**
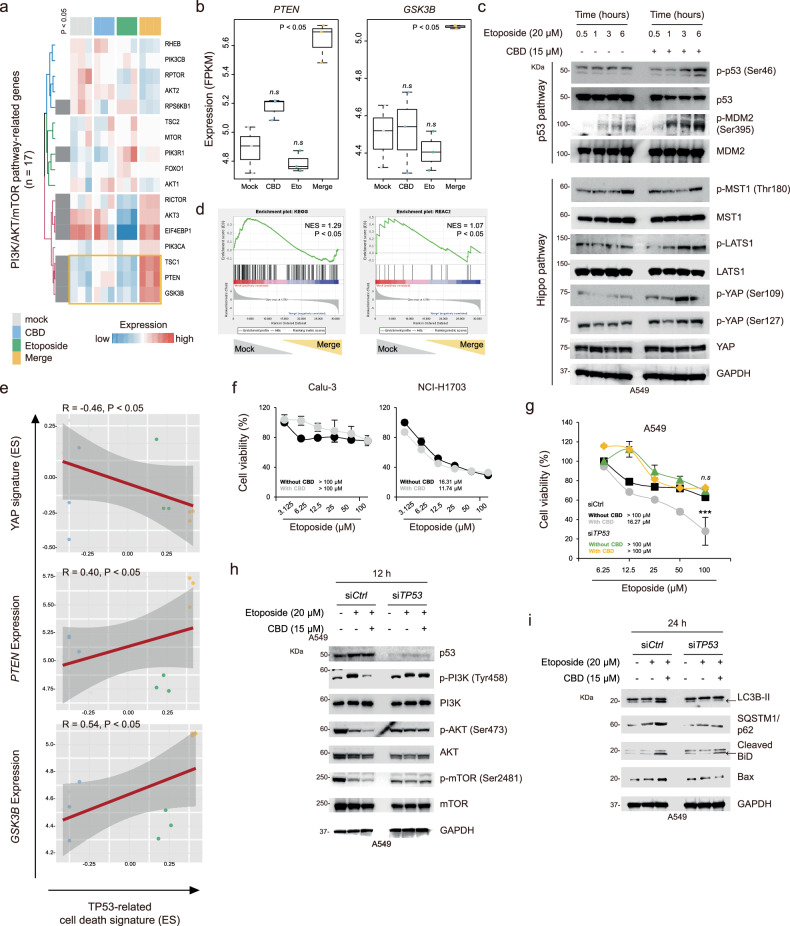
Autophagic cell death induced by combination treatment is associated with the LATS1–p53–PTEN axis. **a** Supervised clustering heat map displaying the PI3K–AKT–mTOR-related genes (*n* = 17) associated with cell death across the groups, with each relevant gene indicated. **b** Box plots showing the expression of *PTEN* and *GSK3B*. Statistical significance is indicated (versus mock; *P* < 0.05; Student’s *t*-test). **c** Western blot analysis of phospho-p53 (Ser46), p53, phospho-MDM2 (Ser395), MDM2, phospho-MST1 (Thr180), MST1, phospho-LATS1 (Thr1079), LATS1, phospho-YAP (Ser109 and Ser127) and YAP, normalized to GAPDH. **d** GSEA results showing the enrichment of mTOR pathway-related signatures (KEGG and REAC). **e** Correlations between p53-related cell death signature and YAP signature, PTEN expression and GSK3B expression. **f** Point plots displaying cell viability at the indicated dose in Calu-3 and NCI-H1703 cells for 48 h. **g** A point plot showing cell viability at the indicated dose in A549 cells treated with control siRNA and siTP53 for 48 h. Statistical significance is indicated (versus siCtrl; ***P < 0.001; Student’s *t*-test). **h**, **i** Western blot analysis of phospho-p53 (Ser46), p53, phospho-PI3K (Tyr458), PI3K, phospho-AKT (Ser473), AKT, phospho-mTOR (Ser2481), mTOR, LC3B, SQSTM1/p62, cleaved BiD and Bax, normalized to GAPDH.

Based on the obtained results, we further analyzed the expression of PI3K–AKT–mTOR-related genes involved in autophagic traits. We observed that the merge groups expressed significantly higher levels of *PTEN*, *GSK3B* and *TSC1* than the other groups (*P* < 0.05; Fig. 5a, b). PTEN and GSK3B, key regulators associated with the inactivation of the mTOR pathway^19,21^, are also involved in the activation of the tumor suppressor protein p53 pathway, which triggers cell death^22,23^. In this study, we demonstrated that the combination treatment with etoposide and CBD enhances the phosphorylation of p53 at Ser46 and MDM2 proto-oncogene at Ser395 in a time-dependent manner, resulting in apoptosis and p53 activation (Fig. 5c, top)^24^. Furthermore, the combination treatment reduced the Hippo pathway-related gene expression, known to be associated with p53 inactivation and cell cycle regulation^25,26^. We also observed increased phosphorylation of large tumor suppressor kinase 1 (LATS1) and yes-associated protein 1 (YAP) but not of macrophage stimulating 1 (MST1) (Fig. 5c, bottom). GSEA also revealed decreased enriched expression of mTOR pathway-related signatures in nontreated cells (*P* < 0.05; Fig. 5d). LATS1 phosphorylation regulates p53 activation^25^, where the associated cell death genes were closely correlated with the expression levels of PTEN and GSK3B (*R* ≥ 0.4, *P* < 0.05) but showed a significant negative correlation with the expression of Hippo pathway-related genes (*R* = −0.46, *P* < 0.05; Fig. 5e). Collectively, our findings suggest that p53 activation is a crucial regulator of autophagic cell death induced by the combination treatment. To evaluate whether the combination treatment is significantly associated with the p53 status, we assessed the synergistic effects of CBD and etoposide on cell viability using p53 mutant cell lines, such as Calu-3 (c.710G>A) and NCI-H1703 (c.853G>A). We found that the viability of these cells was not significantly affected and was comparable to that of the wild-type *TP53* cell line A549 (Fig. 5f)^27^. To further substantiate that the combination treatment specifically targets wild-type *TP53*, we performed additional experiments using p53-null cell lines, NCI-H358 and NCI-H1299 (Supplementary Fig. 5a). Using lentiviral transduction, wild-type *TP53* was stably overexpressed in these cell lines (Supplementary Fig. 5b), and combination treatment was reassessed. Notably, the combination treatment resulted in a notable reduction in cell viability in wild-type *TP53*-overexpressing cells compared with control cells, revealing that the presence of functional wild-type *TP53* enhances the efficacy of combination treatment (Supplementary Fig. 5c). To further validate the effects of p53 after the combination treatment, we conducted TP53 knockdown using siRNA. The viability of cells with suppressed *TP53* expression was not significantly altered after the combination treatment compared with the control group (Fig. 5g).

Further, we assessed the PI3K–AKT–mTOR pathway and found that the downregulation of TP53 expression enhanced the phosphorylation of the key components in the pathway after the combination treatment (Fig. 5h). Furthermore, we found that the synergetic effect on autophagy and cell death markers, such as LC3B-II, p62, cleaved BiD and Bax, was notably reduced by p53 depletion (Fig. 5i). These findings suggest that CBD might enhance etoposide-induced cell death via p53 activation, which in turn regulates the mTOR pathway and subsequently induces autophagic traits.

### Autophagic cell death induced by combination treatment is not associated with the canonical pathways of CNRs and TRPVs

**Fig. 6:**
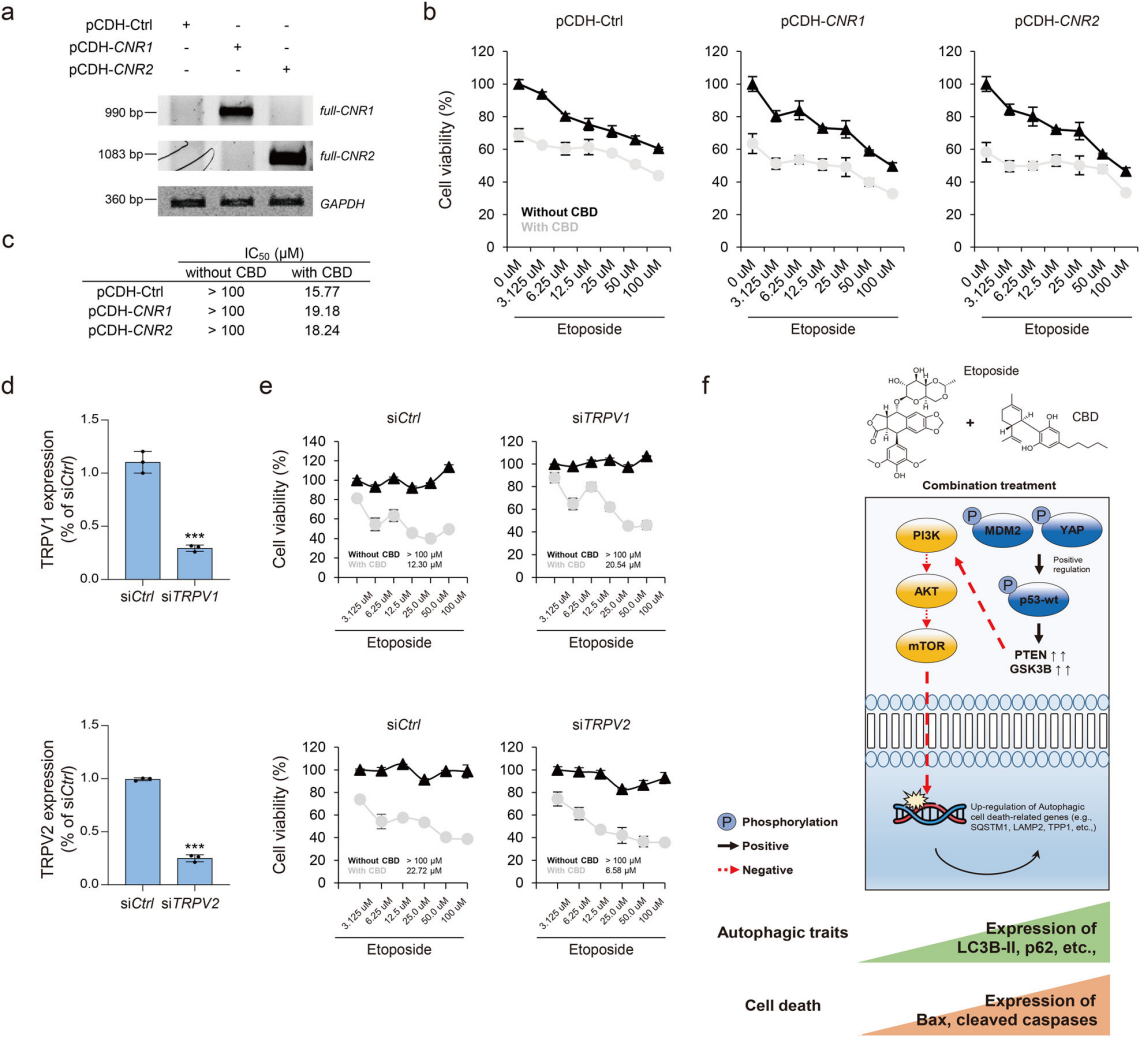
Association between autophagic cell death induced by combination treatment and the canonical pathways of CNRs and TRPVs. **a** Expression levels of *CNR1* and *CNR2* are evaluated by PCR with mRNA amplification normalized to GAPDH. **b** Point plots showing cell viability in A549 cells treated with etoposide, with and without CBD (15 μM), across each stable cell line at various concentrations for 48 h. **c** IC_50_ values of etoposide with and without CBD for each stable cell line. **d** Bar plots display the expression levels of TRPV1 and TRPV2 genes across control siRNA and siTRPV1 and siTRPV2 treatments. Statistical significance is indicated (versus siCtrl; ***P < 0.001; Student’s *t*-test). **e** Point plots showing cell viability in A549 cells treated with etoposide, with and without CBD (15 μM), across various concentrations in each siRNA-treated cell for 48 h. **f** Combination treatment resulting in decreased mTOR pathway and enhanced autophagic cell death.

CBD does not directly bind to CNRs and transient receptor potential cation channel subfamily V members (TRPVs). However, it has powerful indirect effects that are still being studied^28,29^. This study evaluated whether the expression of CNR1 and CNR2 could influence the effects observed with the combination treatment. To this end, we developed CNR overexpression models using A549 cells, which exhibit little endogenous expression of CNR1 and CNR2 (Fig. 6a). As shown in Fig. 6b, c, the inhibition of cell proliferation induced by the combination treatment was independent of CNR1 and CNR2 expression. Furthermore, using siRNA targeting TRPVs such as TRPV1 and TRPV2, we found that TPRVs were also not involved in the synergistic effects induced by the combination treatment (Fig. 6d, e). In summary, the synergistic effects of the combination treatment were independent of both CNR or TRPV signaling pathways, revealing the autophagic cell death.

## Discussion

NSCLC remains a formidable challenge in oncology owing to its genetic heterogeneity and propensity for developing drug resistance. While targeted therapies such as osimertinib and alectinib have shown effectiveness in tumors with specific mutations, such as EGFR and ALK, their utility is confined to these particular genetic alterations, leaving a significant gap in treatment options for patients with other mutations or those who develop resistance to these targeted agents^30,31^.

In this study, we explored the potential of CBD, a nonpsychoactive component of cannabis, to enhance the efficacy of etoposide in treating NSCLC. Our findings demonstrate that the combination of CBD and etoposide significantly promotes apoptosis in NSCLC cells while having minimal effect on normal lung fibroblasts (summarized in Fig. 6f), indicated by the upregulation of key apoptotic markers, including cleaved caspases 8 and 9, PARP and Bax. This is further supported by transcriptomic analyses, which revealed a substantial upregulation of autophagy-related genes such as *SQSTM1*, *LAMP2* and *TPP1*, implicating these pathways in the promotion of autophagic cell death^32–34^. Moreover, proteomic analysis using a multiphospho-kinase array demonstrated that the inactivation of the PI3K–AKT–mTOR signaling pathway is a key driver of the observed effects, aligning with existing literature that underscores the importance of this pathway in cancer cell survival and autophagy regulation^19,20^.

The observed downregulation of critical oncogenes such as *KRAS*, *NRAS* and *MYC* further highlights the potential of this combination therapy to target multiple oncogenic pathways, reducing the likelihood of cancer cell survival and proliferation. This multitargeted approach is particularly advantageous in the context of NSCLC, where oncogene-driven proliferation remains a significant therapeutic challenge.

Importantly, our study elucidates the critical role of the p53 pathway in the efficacy of the combination treatment. The activation of p53, along with the upregulation of *PTEN* and *GSK3B*, appears to be instrumental in mediating the apoptotic and autophagic responses. This finding is particularly relevant given the high frequency of p53 mutations in NSCLC and their association with resistance to conventional therapies. The lack of important effects in p53 mutant cell lines and the results from TP53-knockdown experiments underscore the central role of p53 in the therapeutic mechanism of CBD and etoposide, suggesting that this combination could overcome the limitations of existing therapies in treating p53-mutated NSCLC^22,23^.

Furthermore, our results indicate that the synergistic effects of CBD and etoposide are independent of CNR and TRPV pathways. This finding suggests that CBD may exert its anticancer effects through noncanonical mechanisms, broadening its potential therapeutic applications beyond traditional cannabinoid targets.

In line with the concept of drug repurposing, which has been successfully demonstrated with medications including metformin, thalidomide and aspirin for their anticancer properties, our study suggests that etoposide, a chemotherapeutic agent traditionally used for SCLC, can be effectively repurposed for NSCLC when combined with CBD^35–37^. The enhanced efficacy observed, particularly in the induction of autophagic cell death and suppression of key oncogenic pathways, highlights the potential of this combination therapy as a novel treatment strategy for NSCLC.

In conclusion, the combination of CBD and etoposide presents a compelling therapeutic strategy for NSCLC, leveraging mechanisms of autophagy, apoptosis and oncogene suppression. These findings not only provide a strong rationale for further exploration in preclinical and clinical settings but also suggest the potential to address key challenges in NSCLC treatment, such as drug resistance and the limitations of existing therapies. Furthermore, this combination therapy holds particular promise for patients with p53 mutations or those who have developed resistance to EGFR inhibitors (for example, osimertinib) or ALK-targeted drugs (for example, alectinib), providing a promising alternative approach for improving the outcomes of patients with NSCLC.

## Supplementary information

Supplementary Information

Supplementary Table

## Data Availability

The transcriptome profile data are available via the GEO database at http://www.ncbi.nlm.nih.gov/projects/geo under accession number GSE285498. Additional data generated and analyzed during this study are available from the corresponding author upon reasonable request.
