# Supplementary material for: Cannabidiol potentiates p53-driven autophagic cell death in non-small cell lung cancer following DNA damage: a novel synergistic approach beyond canonical pathways
Source: Exp Mol Med. 2025 May 1;57(5):979–89. doi: 10.1038/s12276-025-01444-x (PMC12130484; doi:10.1038/s12276-025-01444-x)
Supplement: Supplementary file 1 — Supplementary Information [file 12276_2025_1444_MOESM1_ESM.pdf]

Cannabidiol Potentiates p53-Driven Autophagic Cell Death in Non-Small Cell Lung Cancer Following DNA Damage: A Novel Synergistic Approach Beyond Canonical Pathways

Youngsic Jeon[1,†], Taejung Kim[1,2,†], Hyukjoon Kwon[1], Young Nyun Park[3], Tae-Hyung Kwon[4], Min Hong[4], Kyung-Chul Choi[5,*], Jungyeob Ham[1,2,6,*], and Young-Joo Kim[1,*]

Author Affiliations:

[1] Institute of Natural Products, Korea Institute of Science and Technology, Republic of Korea

[2] Natural Product Applied Science, KIST School, University of Science and Technology, Republic of Korea

[3] Department of Pathology, Graduate School of Medical Science, Brain Korea 21 Project, Yonsei University College of Medicine, Republic of Korea

[4] Institute of Biological Resources, Chuncheon Bioindustry Foundation, Republic of Korea

[5] Department of Biochemistry and Molecular Biology, Brain Korea 21 project, Asan Medical Center, University of Ulsan College of Medicine, Republic of Korea

[6] NeoCannBio Co., Ltd., Republic of Korea

†Youngsic Jeon and Taejung Kim contributed equally to this work.

Supplementary Figures

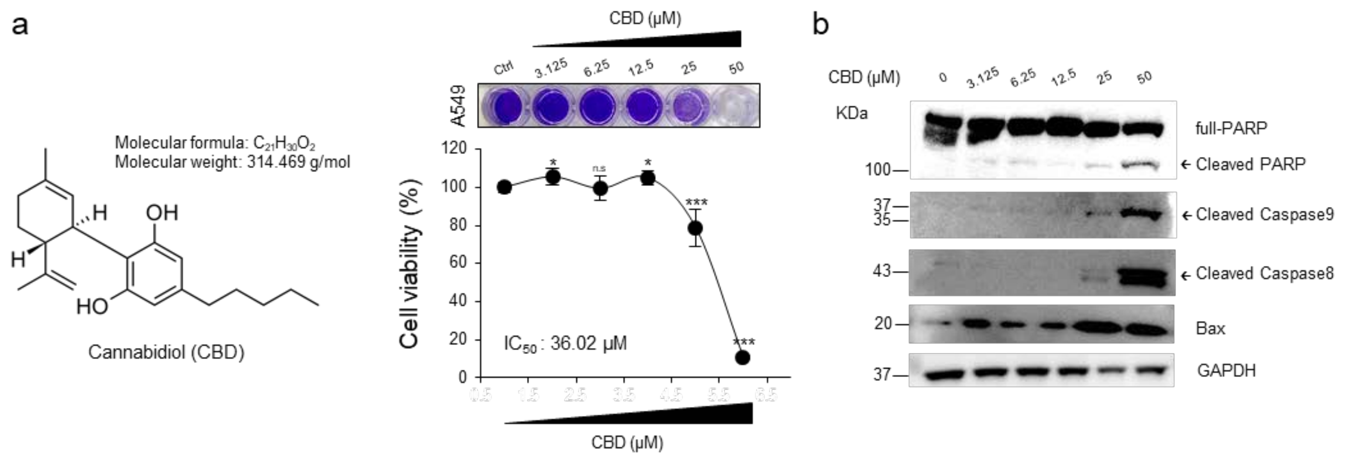

Supplementary Fig. 1 Low concentrations of CBD do not induce cell death in A539 cells. (**a**) Chemical structure of CBD (*left*). Cell viability is shown with a point plot at the indicated dose in A549 cells for 48 h (*right*). (**b**) Expression levels of cleaved PARP, caspases 8 and 9, and Bax are evaluated by Western blot. Protein levels are normalized to those of GAPDH.

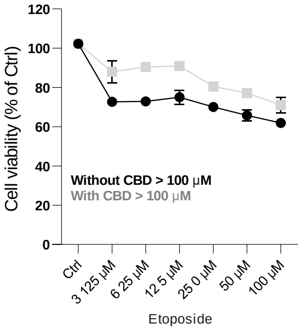

Supplementary Fig. 2 Assessment of combination treatment efficacy using WST-8 assay on normal lung fibroblast cells. A plot shows the cell viability of HEL299 cells treated with etoposide, with or without CBD, for 48 h. IC_{50} values are indicated.

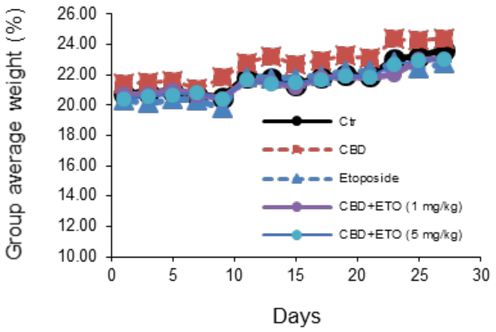

Supplementary Fig. 3 Body weight change during *in vivo* experiment. A plot shows the body weight change during *in vivo* experiment.

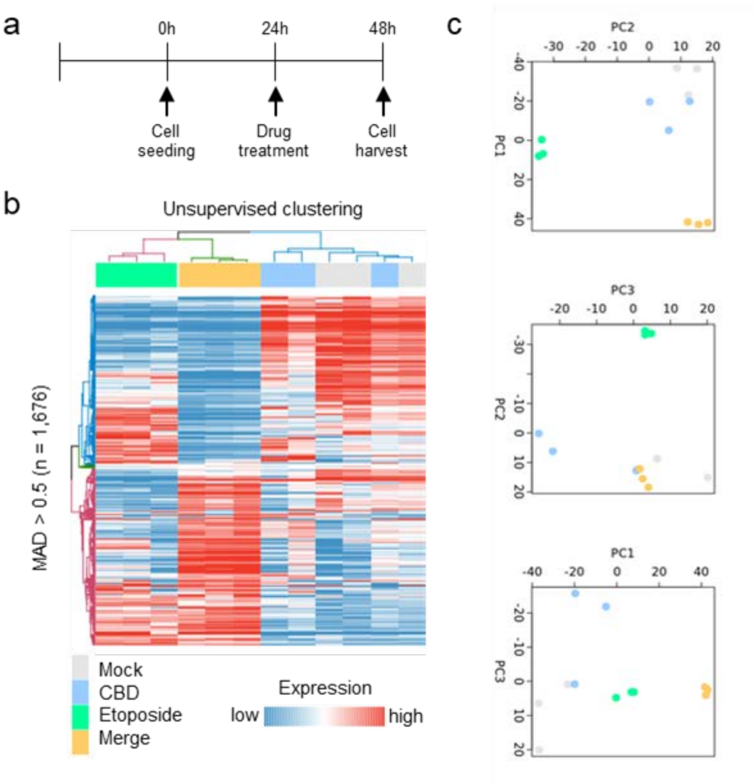

Supplementary Fig. 4 Unsupervised clustering heatmap and PCA. (**a**) The schematic illustrates the workflow and details of sample preparation process of A549 cells for RNA-seq. A549 cells treated with CBD (15 μM), etoposide (20 μM), and their combination (merge) at 24 h. (**b**) A heatmap shows the unsupervised clustering of transcriptome data, demonstrating that the transcriptome of variably expressed genes (median absolute deviation > 0.5, n = 1,676) shows distinct patterns based on treatment status. (**c**) Principal component analysis using these variable genes reveals that groups are notably distributed in distinct clusters.

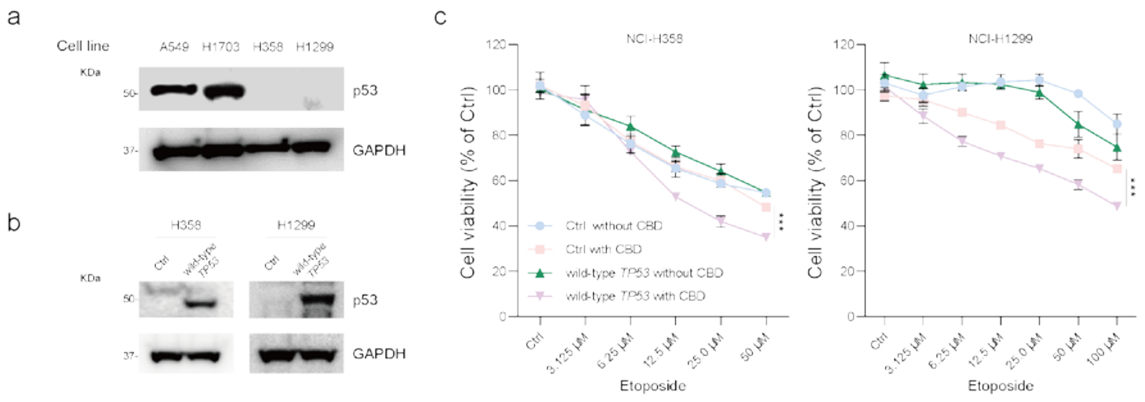

Supplementary Fig. 5 Establishment of p53-overexpressing cell lines and assessment of combination treatment efficacy using WST-8 assay. (**a**) Western blot analysis of p53 and GAPDH in NSCLC cell lines (A549, NCI-H1703, NCI-H358, and NCI-H1299). (**b**) Western blot analysis of p53 and GAPDH in control cells (Ctrl) and established wild-type *TP53*-overexpressing cells. (**c**) Plots show the cell viability of Ctrl and wild-type *TP53*-overexpressing cells treated with etoposide, with or without CBD, for 48 h in NCI-H358 and NCI-H1299, respectively. Statistical significance is indicated (*** $P < 0.001$; Student's t-test).
