# Supplementary material for: Cannabidiol potentiates p53-driven autophagic cell death in non-small cell lung cancer following DNA damage: a novel synergistic approach beyond canonical pathways
Source: Exp Mol Med. 2025 May 1;57(5):979–89. doi: 10.1038/s12276-025-01444-x (PMC12130484; doi:10.1038/s12276-025-01444-x)
Supplement: Supplementary file 2 — Supplementary Table [file 12276_2025_1444_MOESM2_ESM.pdf]

Supplementary Table 1. Antibodies used from western blotting and immunofluorescence

Antibodies	Company	Identifier	Dilution for WB	Dilution for IF
Rabbit monoclonal anti-PARP	Cell Signaling	Cat# 9532; RRID: AB_659884	1:1,000	
Rabbit monoclonal anti-Bax	Cell Signaling	Cat# 41162; RRID: AB_2924730	1:1,000	
Rabbit monoclonal anti-Bcl2	Cell Signaling	Cat# 15071; RRID: AB_2744528	1:1,000	
Rabbit monoclonal anti-LC3B	Cell Signaling	Cat# 3868; RRID: AB_2137707	1:1,000	1:50 - 1:200
Rabbit polyclonal anti-Beclin-1	Cell Signaling	Cat# 3738; RRID: AB_490837	1:1,000	
Rabbit monoclonal anti-SQSTM1/p62	Cell Signaling	Cat# 39749; RRID: AB_2799160	1:1,000	1:50 - 1:200
Rabbit polyclonal anti-BID	Cell Signaling	Cat# 2002; RRID: AB_10692485	1:1,000	
Rabbit polyclonal anti-Cleaved Caspase-9	Cell Signaling	Cat# 9509; RRID: AB_2073476	1:1,000	
Rabbit polyclonal anti-Cleaved Caspase-8	Cell Signaling	Cat# 8592; RRID: AB_10891784	1:1,000	
Rabbit monoclonal anti-Phospho-PI3K (Tyr458)	Cell Signaling	Cat# 17366; RRID: AB_2895293	1:1,000	
Rabbit monoclonal anti-PI3K	Cell Signaling	Cat# 4257; RRID: AB_659889	1:1,000	
Rabbit polyclonal anti-Phospho-AKT (Ser473)	Cell Signaling	Cat# 9271; RRID: AB_329825	1:1,000	
Rabbit monoclonal anti-AKT	Cell Signaling	Cat# 4685; RRID: AB_2225340	1:1,000	
Rabbit polyclonal anti-Phospho-mTOR (Ser2481)	Cell Signaling	Cat# 2974; RRID: AB_2262884	1:1,000	
Rabbit monoclonal anti-mTOR	Cell Signaling	Cat# 2983; RRID: AB_2105622	1:1,000	
Rabbit polyclonal anti-Phospho-p53 (Ser46)	Cell Signaling	Cat# 2521; RRID: AB_10828689	1:1,000	
Rabbit monoclonal anti-p53	Cell Signaling	Cat# 2527; RRID: AB_10695803	1:1,000	
Rabbit polyclonal anti-Phospho-MDM2 (Ser395)	Thermo Fisher	Cat# PA5-13008; RRID: AB_2235259	1:1,000	
Rabbit monoclonal anti-MDM2	Cell Signaling	Cat# 86934; RRID: AB_2784534	1:1,000	
Rabbit monoclonal anti-Phospho-MST1 (Thr180)	Cell Signaling	Cat# 49332; RRID: AB_2799355	1:1,000	
Rabbit polyclonal anti-MST1	Cell Signaling	Cat# 3682; RRID: AB_2144632	1:1,000	
Rabbit monoclonal anti-Phospho-LATS1 (Thr1079)	Cell Signaling	Cat# 8654; RRID: AB_10971635	1:1,000	
Rabbit monoclonal anti-LATS1	Cell Signaling	Cat# 3477; RRID: AB_2133513	1:1,000	
Rabbit monoclonal anti-Phospho-YAP (Ser109)	Cell Signaling	Cat# 53749; RRID: AB_2799445	1:1,000	
Rabbit polyclonal anti-Phospho-YAP (Ser127)	Cell Signaling	Cat# 4911; RRID: AB_2218913	1:1,000	
Rabbit monoclonal anti-YAP	Cell Signaling	Cat# 14074; RRID: AB_2650491	1:1,000	
Rabbit monoclonal anti-GAPDH	Cell Signaling	Cat# 2118; RRID: AB_10698756	1:1,000	
anti-mouse IgG, HRP-linked Antibody	Cell Signaling	Cat# 7074; RRID: AB_2099233	1:2,000	

WB, western blotting; IF, immunofluorescence

Supplementary Table 2. Information on tools and references used

Tool/reference	description	version	Cite
Tophat2	Alignment of transcriptomes using RNA-seq reads	V2. 1. 1	https://ccb.jhu.edu/software/tophat
Cufflinks	Assembly of aligned RNA-Seq reads	V2. 2. 1	https://cole-trapnell-lab.github.io/cufflinks/manual/
R	Software environment for statistical computing and visualization	V4. 2. 2	https://www.r-project.org/
GRCh38.primary_assembly.genome.fa	FASTA file for sequence alignment	Release 47	https://www.gencodegenes.org/human/
Gencode.v45.basic.anontation.2.gtf	GFT file for annotaion	Release 45	https://www.gencodegenes.org/human/release_45.html

Supplementary Table 3. Primers used for qRT-PCR and cloning

Gene name	Purpose	Species	Sequence (5'→3')	Annealing
CNR1 infusion F	Cloning	Human	CATAGAAGATTCTAGATGAAGTCGATCCTAGATGGCC	65°C
CNR1 infusion *R*	Cloning	Human	AGATCCTTCGCGGCCTCACAGAGCCTCGGCAGAC	
CNR2 infusion *F*	Cloning	Human	CATAGAAGATTCTAGATGGAGGAATGCTGGGTGAC	65°C
CNR2 infusion *R*	Cloning	Human	AGATCCTTCGCGGCCTCAGCAATCAGAGAGGTCTAG	
TP53 infusion *F*	Cloning	Human	CATAGAAGATTCTAGATGGAGGAGCCGCAGTCAG	68°C
TP53 infusion *R*	Cloning	Human	AGATCCTTCGCGGCCTCAGTCTGAGTCAGGCCC	
TRPV1 F	qRT-PCR/ PCR	Human	CAAACCGATTTGACCGAGAT	65°C
TRPV1 R	qRT-PCR/ PCR	Human	GTTCAGCACAGCCTTCATCA	
TRPV2 F	qRT-PCR/ PCR	Human	CAAACCGATTTGACCGAGAT	65°C
TRPV2 R	qRT-PCR/ PCR	Human	GTTCAGCACAGCCTTCATCA	
GAPDH F	qRT-PCR/ PCR	Human	CAGCCTCAAGATCATCAGCA	58°C
GAPDH R	qRT-PCR/ PCR	Human	TGTGGTCATGAGTCCTTCCA	

F, forward; R, reverse.

Supplementary Table 4. IC_{50} value of combination treatment with cannabis-related compounds and etoposide

Drug	IC_{50} (μM), 48 h
	with Etoposide
CBDA	> 100
CBD	15.77±1.35
THC	> 100
THCA	> 100

Supplementary Table 5. List of differentially expressed genes (DEGs)

No	Up genes (n = 1,157)	Down genes (n = 1,125)
1	LCN2	HES4
2	IFI6	MXRA8
3	ISG15	MRPL20
4	LCE1F	NPHP4
5	MT1X	ERRFI1
6	IFIT3	CENPS-CORT
7	MX1	CENPS
8	MT2A	EFHD2
9	OASL	AGMAT
10	GALNT12	PADI1
11	CCL5	KLHDC7A
12	IFIT1	RCC2
13	CD68	IFFO2
14	METRNL	MRTO4
15	IL6	CAPZB
16	BCL2A1	MICOS10
17	PI3	RAP1GAP
18	SAA2	ECE1
19	IFIT2	E2F2
20	LAMC2	ID3
21	STC1	SELENON
22	CSF2	AUNIP
23	ANXA10	HMGN2
24	TMEM171	TENT5B
25	IL24	TRNAU1AP
26	IGFN1	ZMYM6
27	ITGB3	CLSPN
28	PLAUR	CDCA8
29	SPOCD1	HEYL
30	CDCP1	CITED4
31	TNFAIP3	EDN2
32	TMEM158	SLFNL1
33	SFN	CDC20
34	HERC5	STIL
35	IL1A	CDKN2C
36	CTSS	ORC1
37	RRAD	PRPF38A
38	HK2	COA7
39	ZFP36	PRKAA2
40	HELZ2	NFIA
41	CLMP	ROR1
42	SLC52A1	DEPDC1

43	HSPA1A	LRRC40
44	HSPA1B	GNG12
45	THBD	LHX8
46	PARP9	CRYZ
47	PRPH	ZZZ3
48	GREM1	ZNHIT6
49	NSG1	HS2ST1
50	PNRC1	LRRC8B
51	GFPT2	CDC7
52	HES2	SLC44A3
53	CSTA	ALG14
54	THEMIS2	TLCD4
55	TIPARP	DBT
56	SLC25A37	COL11A1
57	NKX3-1	VAV3
58	GCOM1	SLC25A24
59	BEX4	CELSR2
60	OAS2	PSRC1
61	ARVCF	SORT1
62	NECTIN4	GSTM4
63	ZNF385A	EPS8L3
64	SP100	AHCYL1
65	FLYWCH1	WDR77
66	MARCHF3	NRAS
67	STAT2	PTGFRN
68	CHST1	MAN1A2
69	CRTAC1	WDR3
70	BEX2	FAM72B
71	YPEL3	FAM72C
72	THSD1	FAM72D
73	IRAK2	PEX11B
74	UNC5B	H2AC20
75	ZC3H12A	ANP32E
76	CDKN1A	TUFT1
77	TM4SF19	S100A10
78	SCG5	S100A4
79	DNAJB5	S100A3
80	PML	CKS1B
81	ITPR3	TAGLN2
82	NGFR	UCK2
83	BST2	MPC2
84	ARG2	CENPL
85	COL6A2	RASAL2
86	MUSTN1	KIF14

87	IFIH1	CFH
88	KDELR3	CSRP1
89	UBE2L6	UBE2T
90	GPX1	SNRPE
91	ABLIM3	NUAK2
92	HMGA2	NUCKS1
93	PLXNB3	KCNH1
94	PARP12	NEK2
95	BMF	KCTD3
96	N4BP3	RRP15
97	WBP2	TGFB2
98	TIMP4	IARS2
99	REC8	MTARC1
100	TMOD1	ENAH
101	STRIP2	SRP9
102	SLAMF7	H3-3A
103	GALNT9	ABCB10
104	LYNX1	URB2
105	RNF19B	FAM89A
106	HSPA6	CHML
107	CALCOCO1	TBCE
108	NR0B1	COX20
109	AREG	TFB2M
110	TIMP1	GTPBP4
111	PLEKHM1	IDI2
112	GDF15	ZMYND11
113	PPP1R18	ANKRD16
114	MT1A	ITIH2
115	SH2D2A	PFKFB3
116	C1QTNF1	MCM10
117	GCH1	HACD1
118	ARC	BMI1
119	DRAXIN	MSRB2
120	TNFRSF10D	PDSS1
121	LPIN3	MASTL
122	MILR1	ANKRD30A
123	SH3KBP1	PARD3
124	SP140L	GPRIN2
125	PARP14	NPY4R
126	XDH	ZNF488
127	HMGA1	SYT15B
128	FCHSD1	NPY4R2
129	CLGN	A1CF
130	DHRS7	ZWINT

131	ATG16L2	IPMK
132	GK	CISD1
133	PLA2G4C	BICC1
134	ULBP2	RHOBTB1
135	LZTS3	RTKN2
136	NAMPT	ARID5B
137	LY96	PBLD
138	HSD17B14	HKDC1
139	DHRS9	TSPAN15
140	DTX3L	DDX21
141	FBXO32	VSIR
142	TNFSF9	NUDT13
143	GRIP2	PAPSS2
144	SERTAD1	KIF20B
145	ARHGEF4	ANKRD1
146	BTN3A1	PPP1R3C
147	ZC3HAV1	CYP26A1
148	G0S2	KIF11
149	TP53INP1	CEP55
150	SAMD9	PLCE1
151	LAT	ANKRD2
152	RIN1	PGAM1
153	POLR2J2	AVPI1
154	PLK3	CNNM1
155	BMAL2	CPN1
156	SMIM29	ERLIN1
157	ST6GALNAC4	NOLC1
158	NIPAL4	TRUB1
159	TRIM21	PRDX3
160	CTSL	TACC2
161	ZNFX1	MKI67
162	S100P	ECHS1
163	ASTN2	MTG1
164	FDXR	ZNF511
165	DUSP5	TSPAN4
166	SPRR2D	CARS1
167	CNNM4	TRIM6
168	EVI2B	FAR1
169	PTGS2	TEAD1
170	SLFN5	E2F8
171	LACC1	KIF18A
172	ENGASE	LGR4
173	DMKN	IMMP1L
174	P2RX4	BDNF

175	BDKRB1	ELP4
176	ICOSLG	CAT
177	GNRH1	FJX1
178	TYMP	ALX4
179	APOL6	ARFGAP2
180	SERINC2	ACP2
181	PLPP5	CKAP5
182	FADS3	P2RX3
183	L1CAM	TIMM10
184	TPST2	RTN4RL2
185	MERTK	FAM111B
186	SAT1	FEN1
187	NPTXR	INCENP
188	IER3	ASRGL1
189	GOLGA8A	GANAB
190	WHRN	RCOR2
191	RAB38	COX8A
192	TMEM52	FLRT1
193	IDUA	BANF1
194	YPEL4	PACS1
195	DYSF	RBM14
196	TNFRSF25	RBM4
197	ZMAT3	PC
198	PGF	TPCN2
199	ADAMTS7	SHANK2
200	SLC17A5	XNDC1N
201	SAMD9L	ARHGEF17
202	PLXNA3	DNAJB13
203	MICALL1	UCP2
204	RNF24	LIPT2
205	CFAP36	LRRC51
206	LRP1	ANAPC15
207	H2BC5	ARRB1
208	GPCPD1	SYTL2
209	HCAR2	ARHGAP42
210	IFI44L	PDGFD
211	NTN1	SLN
212	ASS1	ALKBH8
213	CACNG8	PIH1D2
214	TNFAIP8L3	TIMM8B
215	TPBG	H2AX
216	LRRC75A	SORL1
217	TMEM92	GRAMD1B
218	ABHD17C	NRGN

219	EEIG1	SPA17
220	TSPYL2	CHEK1
221	EML2	HYLS1
222	CREBRF	NCAPD3
223	MROH1	FOXM1
224	TRIB1	TEAD4
225	CORO2B	TPI1
226	VDR	MAGOHB
227	H2AC18	LRP6
228	H2AC19	PDE3A
229	FBXO44	SLCO1B1
230	TMCC3	ETNK1
231	RGMA	ETFRF1
232	LYNX1-SLURP2	KRAS
233	SH3BGRL3	FGD4
234	TPRG1L	PKP2
235	GAMT	YAF2
236	MYO5A	PUS7L
237	TMEM59L	CNTN1
238	BORCS5	SLC38A2
239	BLOC1S5-TXNDC5	PCED1B
240	MAGED4	TROAP
241	PRKD2	KRT80
242	DNAJA1	KRT4
243	TMEM132A	KRT81
244	BTN3A2	ESPL1
245	NPIPB11	TNS2
246	IFI35	HOXC8
247	MAPRE3	TMT1B
248	TPP1	BLOC1S1
249	BICDL1	RDH5
250	ATG2A	KRT18
251	RRM2B	PA2G4
252	MDM2	RBMS2
253	MT1G	ATP5F1B
254	MAGED4B	GLI1
255	SPEG	YEATS4
256	SP110	LIN7A
257	ATP6V1F	ALX1
258	ACER2	RASSF9
259	ARSG	NTS
260	RRBP1	GALNT4
261	MICAL1	SNRPF
262	EFNB1	GAS2L3

263	HPS3	UTP20
264	DUSP6	PARPBP
265	CASP10	PMCH
266	HERC6	SLC25A3
267	LCE1E	NFYB
268	HRH1	NUAK1
269	ELL2	CRY1
270	USF1	ABTB3
271	SQOR	IFT81
272	SPATA18	SDSL
273	BEST1	PEBP1
274	MAGEH1	CIT
275	ALOX5	HNF1A
276	BRICD5	KMT5A
277	PLEKHO2	GTF2H3
278	GIPR	NCOR2
279	CSNK1E	PUS1
280	INSYN2B	PXMP2
281	AK1	ZNF605
282	KDM7A	LATS2
283	SLC26A6	SKA3
284	FNDC4	MIPEP
285	NBPF15	MTMR6
286	PSMB8	SLC7A1
287	ADAR	BRCA2
288	NBPF9	HMGB1
289	IL33	STARD13
290	CGB7	RFC3
291	ADRB2	POSTN
292	LAPTM5	VWA8
293	AADAC	GPALPP1
294	FRMD8	NUDT15
295	PLA2G4B	EBPL
296	ANKRD29	SPRYD7
297	MIA3	DIAPH3
298	H2BC12	PCDH9
299	SHC2	MZT1
300	TTC39B	BORA
301	ASAH1	KLF5
302	TDRD7	NDFIP2
303	USP18	LMO7
304	VWCE	GPC6
305	CCN3	MBNL2
306	F3	NALCN

307	RTL5	ITGBL1
308	RRAGD	IRS2
309	SAXO4	COL4A1
310	SRGN	F7
311	NPAS2	ATP11A
312	ACTA2	F10
313	RINL	PCID2
314	DNER	AJUBA
315	H2BC21	DHRS4
316	RAB5B	DHRS4L2
317	CLP1	STXBP6
318	EFR3B	G2E3
319	RPS27	SPTSSA
320	POMGNT1	FANCM
321	CYGB	LRR1
322	RALGDS	POLE2
323	RAB4B	TRIM9
324	CCNO	WDHD1
325	CES2	SPTB
326	TAP1	MTHFD1
327	ATP2B4	ACOT1
328	RAP2B	JDP2
329	SERPINB8	POMT2
330	TRIM3	SPTLC2
331	BHLHE40	NRXN3
332	PIM2	FLRT2
333	SHISAL1	EFCAB11
334	SPRED3	SERPINA3
335	MGST2	CCDC85C
336	TRIM8	EXOC3L4
337	OPTN	TRMT61A
338	GSAP	XRCC3
339	RGS16	CDCA4
340	NRBP2	CRIP1
341	SYT7	TEDC1
342	LIMK1	UBE3A
343	INO80B	GABRB3
344	HOXC4	GABRA5
345	SDC4	TJP1
346	TPRA1	ARHGAP11A
347	BVES	ZNF770
348	GPX3	SPRED1
349	FAM25A	THBS1
350	DCUN1D3	KNL1

351	MFSD2A	INO80
352	EMILIN3	CKMT1A
353	CPPED1	WDR76
354	GPR87	NUSAP1
355	COL16A1	OIP5
356	BCL10	SORD
357	TNFAIP8	DUT
358	BBLN	SLC27A2
359	RBPMS2	MYO5C
360	STN1	PIF1
361	SIN3B	DIS3L
362	TMEM91	SMAD6
363	ARSA	ITGA11
364	MTURN	KIF23
365	GDI1	ZWILCH
366	KCNK6	THSD4
367	TRANK1	NPTN
368	ZNF697	CHRNA5
369	TIFA	PDE8A
370	NAPB	NTRK3
371	TAB3	FANCI
372	ZNF774	BLM
373	RELA	RCCD1
374	LY6K	PRC1
375	STK10	NR2F2
376	CALB2	IGF1R
377	ARL4D	POLR3K
378	KLHDC7B	SNRNP25
379	NOP53	METRN
380	NATD1	MEIOB
381	HLA-E	TEDC2
382	DAXX	VASN
383	QSOX1	EEF2KMT
384	GABBR1	ZC3H7A
385	C1QL4	COQ7
386	MTSS1	ERI2
387	MYPOP	EARS2
388	STMN4	SLC5A11
389	VPS11	QPRT
390	ISY1-RAB43	KIF22
391	CCDC167	DCTPP1
392	AIFM2	SHCBP1
393	SESN1	RPGRIP1L
394	ENTPD4	CDH16

395	GJB2	CDH1
396	INSYN2A	CHTF8
397	ALOXE3	PDF
398	PGGHG	CYB5B
399	CCL3L3	NQO1
400	XAGE1A	MTSS2
401	ZNF581	DHODH
402	MSC	AARS1
403	MDK	BCO1
404	TVP23C-CDRT4	CMIP
405	SLC17A7	CMC2
406	CHST2	GCSH
407	MARCO	CENPN
408	CTSH	COTL1
409	AOC2	CDT1
410	FOSB	DPEP1
411	FOS	SLC7A5
412	MINK1	RFLNB
413	PTPRU	NXN
414	MCRIP1	SERPINF2
415	ZNF574	ITGAE
416	IFNAR2	HASPIN
417	TNIK	UBE2G1
418	RASSF5	ZNF594
419	PTAFR	DHX33
420	GPRIN1	PIMREG
421	CYTH1	ASGR1
422	SMG8	NUP88
423	XAGE1B	EIF5A
424	ARL1	TP53
425	PHLDA2	CYB5D1
426	PPIF	GLP2R
427	DMRTA1	TRIM16
428	CUL7	PMP22
429	TTLL4	SHMT1
430	PPM1K	TMEM97
431	SH2B3	ATAD5
432	COL7A1	CCL2
433	RILPL1	RDM1
434	INPP1	PIGW
435	HCAR3	ACACA
436	GOLGA3	ARHGAP23
437	TMEM151A	CISD3
438	MOB3C	CDC6

439	EFNA4	RARA
440	FXYD5	TOP2A
441	TRAF1	IGFBP4
442	BCL2L2-PABPN1	CAVIN1
443	RSAD2	NAGLU
444	MAGED2	PSMC3IP
445	LIN7B	PLEKHH3
446	VIM	CNTNAP1
447	ANKMY2	FZD2
448	EFL1	DBF4B
449	CYTH2	UBTF
450	LIF	KIF18B
451	SNX21	BRCA1
452	NBPF8	HEXIM1
453	WNT9A	MAP3K14
454	SLC22A15	PRR15L
455	PPY	GIP
456	DIRAS1	SPOP
457	SELENOM	EME1
458	HECA	HLF
459	UBXN7	CUEDC1
460	RRAGA	NME1
461	DEFB1	MKS1
462	H1-2	BRIP1
463	PLEKHF1	PRKCA
464	FAM43A	RAB37
465	CDKN1C	SMIM6
466	RPL36	CASKIN2
467	CYP1A1	MGAT5B
468	PSMB6	TK1
469	SSR3	BIRC5
470	GOLGA2	EIF4A3
471	LAMB3	MRPL12
472	USO1	SLC25A10
473	ITGA5	ARL16
474	GPR108	ALYREF
475	JUND	TYMS
476	MEGF6	ENOSF1
477	ARID3B	NDC80
478	NEURL3	MTCL1
479	CLMN	AFG3L2
480	USP35	CABLES1
481	ZBTB7A	DSC2
482	GMIP	TTR

483	KLHL21	RNF138
484	FAM89B	ACTG1
485	STXBP1	MAPRE2
486	CNP	SMAD7
487	WASHC2A	CTIF
488	CRABP2	SMAD2
489	ROBO3	SKA1
490	CEP170B	ALPK2
491	ZNF337	NEDD4L
492	SCX	PHLPP1
493	CCDC30	BCL2
494	APOL2	TMX3
495	TREX1	SOCS6
496	FAM219A	CYB5A
497	PANX1	ZNF236
498	MARF1	MISP
499	DCP1B	PTBP1
500	PAX8	POLR2E
501	NPIPB12	ATP5F1D
502	CSGALNACT2	MBD3
503	RDH10	TIMM13
504	BPGM	TLE2
505	WTAP	TEKTIP1
506	UQCR11	SEMA6B
507	HAS3	UHRF1
508	EHD3	CRB3
509	ATOSB	VAV1
510	SUSD6	PEX11G
511	IFFO1	ARHGEF18
512	SH3PXD2A	CD320
513	PPP1R14C	HNRNPM
514	ARFGAP1	OLFM2
515	SIX1	PPAN
516	HK1	AP1M2
517	NOVA1	RNASEH2A
518	SCHIP1	ADGRL1
519	MYL5	DDX39A
520	ERFL	GIPC1
521	CCNL1	CYP4F3
522	UBXN1	HAUS8
523	NRBF2	ARRDC2
524	TICAM1	ISYNA1
525	SFRP1	UQCRFS1
526	IGBP1	ZNF507

527	ZMIZ2	NUDT19
528	OGFR	RHPN2
529	TRIML2	ZNF599
530	TNFAIP1	PRODH2
531	CARD6	IGFLR1
532	RTL8C	ZNF260
533	LPAR2	ZNF146
534	HYPK	SARS2
535	STX12	ACTN4
536	RPL12	FCGBP
537	EIF1AD	TMEM145
538	ZFYVE19	LIPE
539	ZNF841	BCAM
540	HLA-A	ZC3H4
541	DYNC1I2	SLC1A5
542	SLC31A1	SYNGR4
543	CDK5R2	SULT2B1
544	RNF168	CA11
545	MAP2	DBP
546	MLLT11	RUVBL2
547	PCDHGC3	ETFB
548	PATL1	CLDND2
549	ENTPD7	TMEM190
550	FIG4	IL11
551	NSMF	ZNF772
552	TOX2	UBE2S
553	CREG1	ID2
554	HDHD3	TTC32
555	GPKOW	MATN3
556	PRX	WDR35
557	MXI1	GEN1
558	TOM1	RAB10
559	GRINA	CAD
560	EIF5A2	WDR43
561	SLC9A7	LBH
562	ZSWIM8	CRIM1
563	TRIM11	SRSF7
564	TMEM225B	EML4
565	APOBEC3C	SLC3A1
566	ZNRF1	LRPPRC
567	TRIM38	CALM2
568	PANX2	CHAC2
569	GRIN3B	SPTBN1
570	NINJ1	PRADC1

571	GPR176	TET3
572	OAF	POLR1A
573	CHURC1	MRPL35
574	RNF39	ST3GAL5
575	WASHC2C	KRCC1
576	COG1	NCAPH
577	TNF	COA5
578	TAOK3	ST6GAL2
579	AGAP5	MALL
580	FBXL20	BUB1
581	ANKRD24	POLR1B
582	MACO1	DDX18
583	SPAG7	TMEM177
584	GUCD1	INHBB
585	FST	EPB41L5
586	TRIM34	MAP3K2
587	TMEM102	GPR39
588	DUSP10	LYPD1
589	IL15RA	NCKAP5
590	FAM167B	MCM6
591	INSL3	ARL6IP6
592	TBC1D17	PKP4
593	GRIN2D	ITGB6
594	PLEKHB2	PSMD14
595	GATA2	FIGN
596	AFAP1L1	GRB14
597	OVCA2	NOSTRIN
598	HSPH1	PHOSPHO2
599	ARPC1B	CDCA7
600	ZNF30	ATP5MC3
601	PLCG2	COL3A1
602	RPL36A-HNRNPH2	OSGEPL1
603	VPS9D1	NEMP2
604	FAM131A	ITGAV
605	TEP1	CAVIN2
606	CASP7	HSPD1
607	SPMIP1	MOB4
608	DUSP7	AGPS
609	TCEAL9	NFE2L2
610	FUT3	FZD5
611	SMURF2	RPE
612	IRGQ	BARD1
613	ITPKC	CPS1
614	ZNF133	USP37

615	MANF	CTDSP1
616	PRMT2	ABCB6
617	RAD52	GLB1L
618	TCAP	TUBA4B
619	ADGRE1	FARSB
620	NBPF19	FN1
621	HINFP	SCG2
622	IFNAR1	IRS1
623	NPIPA3	USP40
624	LCE1B	UGT1A8
625	CYP4F11	UGT1A9
626	DLGAP4	UGT1A6
627	PTEN	SH3BP4
628	NECTIN1	SEPTIN2
629	SAMD4B	SNRPB
630	UBALD2	AP5S1
631	LCE1C	CDC25B
632	ETV3	MCM8
633	NR2C1	CRLS1
634	SEC24A	JAG1
635	ELF3	PLCB4
636	TM2D1	DSTN
637	SBK1	NAA20
638	TNIP2	GZF1
639	ZNF853	FOXS1
640	STX16	TPX2
641	GGA3	E2F1
642	RBM38	PXMP4
643	AKAP17A	MMP24
644	TRIP10	DSN1
645	NPIPA2	TGIF2
646	FAM171B	RBL1
647	GPR3	MANBAL
648	PHF13	LBP
649	HSPBAP1	FAM83D
650	UBE2Z	MYBL2
651	PLEKHM2	HNF4A
652	TMEM63A	SRSF6
653	FBXW7	RIMS4
654	MAP3K5	UBE2C
655	PI4KB	EYA2
656	OSR1	PARD6B
657	REEP2	PEDS1-UBE2V1
658	GOLGA5	NFATC2

659	SEPTIN3	ADNP
660	POM121C	AURKA
661	PMAIP1	RAE1
662	ZBTB34	VAPB
663	HEBP1	CYP24A1
664	RGCC	NKAIN4
665	RPL39	ZNF512B
666	TMEM8B	ARFRP1
667	TMEM44	GABPA
668	ZDHHC4	URB1
669	AGO2	CHAF1B
670	STAT5A	SIM2
671	OTUD5	PSMG1
672	NR1D1	RIPK4
673	LRRC32	U2AF1
674	ARPC1A	HSF2BP
675	BAK1	PDXK
676	SNX12	COL18A1
677	MSANTD3	MICAL3
678	NBPF26	CLTCL1
679	MEGF11	CDC45
680	CCDC71L	TXNRD2
681	RABAC1	RSPH14
682	RELT	SMARCB1
683	EID2B	SUSD2
684	CALU	ZNRF3
685	RNF25	MTFP1
686	TLCD5	PES1
687	GPAT3	TST
688	IFIT5	CDC42EP1
689	RAB6A	H1-0
690	CASK	ANKRD54
691	SNPH	APOBEC3B
692	RASA2	PDGFB
693	TEAD3	DESI1
694	ZNF646	MRTFA
695	RELB	POLR3H
696	HDAC10	CENPM
697	TREM2	XRCC6
698	TNFRSF11A	TTLL12
699	PRODH	RIBC2
700	HMGXB3	GTSE1
701	FAM171A2	CELSR1
702	CD63	CHL1

703	NFATC2IP	LMCD1
704	GID4	CIDEC
705	RASSF8	NUP210
706	CABLES2	OXNAD1
707	CEBPB	KAT2B
708	RAI14	SGO1
709	DPYSL4	ANKRD28
710	PAGE2B	TGFBR2
711	SMTNL1	CNOT10
712	PPP3CC	CLASP2
713	RPL23A	CTDSPL
714	WIPI2	NKIRAS1
715	CATSPERG	NR1D2
716	CHST15	SEC22C
717	CNNM2	KIF9
718	SEC24C	ELP6
719	SMIM38	CDC25A
720	CFAP68	TRAIP
721	GAS6	MST1R
722	SNAI3	DAG1
723	CYBRD1	IFRD2
724	POLH	POC1A
725	ARRDC3	TNNC1
726	TMEM87B	RFT1
727	POLL	CACNA1D
728	SAP130	ACOX2
729	LENG1	THOC7
730	F2RL2	MITF
731	OTUD3	RYBP
732	STBD1	CADM2
733	TMEM243	ARL13B
734	BAHD1	PROS1
735	PITPNC1	RIOX2
736	IRF2BPL	ALCAM
737	HHAT	SIDT1
738	CCDC28A	PHLDB2
739	RPS10-NUDT3	IGSF11
740	WDR6	UPK1B
741	PPARD	B4GALT4
742	CD83	POLQ
743	GTF2E2	HEG1
744	RASGRF2	CHST13
745	TUT7	H1-10
746	IFNL1	MRPL3

747	RHOC	AMOTL2
748	RHOG	TM4SF4
749	ZC3H12C	PFN2
750	SUGCT	SUCNR1
751	GOLGA6L9	GFM1
752	UBAP1	TRIM59-IFT80
753	HSPA12A	ECT2
754	TVP23C	ACTL6A
755	PARP3	MCCC1
756	PLTP	PARL
757	CASP1	TRA2B
758	SAR1A	EIF4G1
759	ABCA1	P3H2
760	FAM193B	LPP
761	HOMER1	MB21D2
762	MAP3K10	CLDN1
763	CCDC85B	LETM1
764	SLC6A15	NSD2
765	SLC6A17	STK32B
766	RPL26	AFAP1
767	WHAMM	CC2D2A
768	KHNYN	NCAPG
769	ZFYVE1	KLB
770	NXF1	SHISA3
771	COG5	CEP135
772	IER3IP1	LIMCH1
773	APBB1IP	PAICS
774	ST7	IGFBP7
775	ZBTB4	UGT2B15
776	GIGYF1	YTHDC1
777	LURAP1L	UGT2B11
778	CSNK2A2	SLC4A4
779	SCAMP2	NAAA
780	ARNT	MRPL1
781	POM121	FRAS1
782	PHYKPL	PLAC8
783	SMTNL2	ABCG2
784	NPPC	TIGD2
785	CDIP1	GPRIN3
786	TMEM38A	ARHGAP24
787	TMEM150C	MAPK10
788	ALKBH7	BMPR1B
789	PCDHAC2	PDLIM5
790	ZUP1	TRMT10A

791	ITPRIP	CENPE
792	GOLGA8O	GAR1
793	ZCCHC24	FAM241A
794	CORO1B	SYNPO2
795	DDRGK1	ANK2
796	UAP1	CCNA2
797	TTYH3	FGF2
798	IFNLR1	PLK4
799	SP6	PGRMC2
800	PANK1	JADE1
801	MSX1	NOCT
802	MT1B	SLC7A11
803	SLC8A1	MAML3
804	TANC2	SCOC
805	CTNS	IL15
806	SEC61B	HHIP
807	PCNX2	FGB
808	YBEY	PLRG1
809	FHDC1	FGA
810	ELOVL3	MND1
811	ANGPTL6	TMEM192
812	TP53INP2	PALLD
813	PI4K2A	GALNT7
814	UBA7	NEIL3
815	LONRF3	WWC2
816	SDCBP2	CLDN22
817	IFITM10	CLPTM1L
818	RPL37	MYO10
819	TMEM63B	CDH6
820	PTPDC1	LMBRD2
821	APBA3	MCIDAS
822	NYAP1	SREK1IP1
823	TMEM255B	CENPK
824	POPDC3	CENPH
825	MAX	ERBIN
826	FOXO4	PTCD2
827	FYN	F2R
828	TIGD3	PDE8B
829	RPL36A	ZBED3
830	CCDC174	DHFR
831	ZNF761	XRCC4
832	DUSP16	NR2F1
833	C1S	GLRX
834	TATDN2	ST8SIA4

835	CIB1	GIN1
836	EBF4	MACIR
837	HDAC9	FBXL17
838	EIF1B	FBN2
839	IL17RD	SLC12A2
840	EPS8L1	UQCRQ
841	PRAF2	FSTL4
842	PHLDA1	CDC25C
843	CDC37L1	TGFBI
844	KIZ	CD14
845	CDC42EP2	NR3C1
846	USP27X	RBM27
847	TSPAN5	SYNPO
848	PUS3	GALNT10
849	TBC1D25	SGCD
850	KAZN	UBLCP1
851	MICA	PTTG1
852	RHBDL2	RNF145
853	UBE2H	WWC1
854	SUN3	SLIT3
855	ETV6	DUSP1
856	PRR13	DRD1
857	GRB10	CPEB4
858	MSX2	CPLX2
859	TMUB2	NOP16
860	MTMR4	FGFR4
861	SLC39A13	HNRNPAB
862	MTFMT	TRIM7
863	TREM1	GMDS
864	LMOD1	SERPINB9
865	SRXN1	RPP40
866	BTBD10	PAK1IP1
867	HABP4	TXNDC5
868	CHMP3	ELOVL2
869	HECTD3	EDN1
870	SORCS2	NEDD9
871	ZG16B	RBM24
872	CSF2RA	DCDC2
873	RPL28	GMNN
874	RUSC1	SLC17A1
875	MRPL17	H4C1
876	NSMAF	H3C2
877	GRHL3	HFE
878	LYST	H1-3

879	NGLY1	NRM
880	MSS51	TCF19
881	UBOX5	DDX39B
882	PIP4K2C	VARS1
883	FUT8	ZBTB12
884	SLC22A17	KIFC1
885	UBE2J1	SPDEF
886	MICB	BLTP3A
887	ISL2	FKBP5
888	HLA-G	KCNK5
889	BCL6	UNC5CL
890	ANKRD10	TFEB
891	OLFM1	BYSL
892	MEIS3	CCND3
893	KLK4	DLK2
894	MAP3K3	CD2AP
895	TTC3	MCM3
896	XYLT2	TMEM14A
897	ZNF565	TRAM2
898	CNOT4	COL21A1
899	MCTS2	FKBP1C
900	CLDND1	KCNQ5
901	LRRC8A	FILIP1
902	SLC25A6	HMGN3
903	MOCOS	TTK
904	ZNF205	ME1
905	TRAF6	AKIRIN2
906	TMEM184B	NDUFAF4
907	ADCY1	COQ3
908	IGDCC4	PREP
909	SPAG9	RTN4IP1
910	GLTP	GOPC
911	PPP1R26	ASF1A
912	RIMS3	HINT3
913	WFS1	NCOA7
914	HEBP2	ECHDC1
915	GCKR	CENPW
916	SCO2	ARHGAP18
917	SDC3	EPB41L2
918	PLEKHA4	CCN2
919	IQCD	PDE7B
920	UQCRB	BCLAF1
921	SRSF5	PHACTR2
922	FAM241B	ADGRG6

923	POMGNT2	UTRN
924	YIF1A	ULBP1
925	TBX19	LRP11
926	UBQLN4	ARMT1
927	ZNF526	AKAP12
928	ABCA3	CNKSR3
929	DCDC2C	ARID1B
930	CPSF4	SLC22A3
931	TRADD	PLG
932	PHETA1	DNAAF5
933	RPL11	MAFK
934	EFCAB10	AIMP2
935	RNASEL	ZNF12
936	GOLGA6L10	RPA3
937	VPS18	THSD7A
938	RASA4B	AGMO
939	BIRC3	POLR1F
940	LMO2	TRA2A
941	NCEH1	CYCS
942	SMIM10L2A	ACTB
943	SOX4	CBX3
944	SNAP29	LSM5
945	NBPF11	ANLN
946	DAP	ELMO1
947	TMEM115	DBNL
948	ARAP1	H2AZ2
949	TUBGCP6	DDC
950	HSPA4L	FIGNL1
951	YJU2B	TNS3
952	E2F7	LANCL2
953	TMEM170A	EGFR
954	VRK3	CCT6A
955	RMC1	VKORC1L1
956	IGFBP6	RFC2
957	UBE2D4	GTF2IRD1
958	ATP2A1	CASTOR2
959	MRPL34	GTF2I
960	GRHL1	UPK3B
961	TTC39C	YWHAG
962	KRTAP5-1	PHTF2
963	AGAP9	CACNA2D1
964	MED12	SEMA3E
965	LHX3	SEMA3C
966	ZNF436	FAM133B

967	ZBTB42	CDK6
968	NMNAT2	GNG11
969	TBC1D3L	PON3
970	ZNF296	PDK4
971	GOLGA8R	SLC25A13
972	ZDHHC18	PON2
973	NHLRC4	PEG10
974	UPK3BL2	ZNF789
975	MAMSTR	MCM7
976	DTX3	NAPEPLD
977	PIP4K2A	ORC5
978	IRF4	LAMB1
979	IRAK1	CAV1
980	ITSN2	GPR37
981	DGKE	NDUFA5
982	EIF4E2	HILPDA
983	KRTCAP3	AKR1B10
984	EZH1	LUC7L2
985	CSNK1G1	TMEM139
986	TPD52L2	ZNF467
987	ZRANB1	ABCB8
988	SLX1B	XRCC2
989	TNC	NCAPG2
990	RPS6KC1	AGPAT5
991	ALAS1	PRAG1
992	STXBP3	SGCZ
993	GCNA	FGL1
994	BTN2A2	MTUS1
995	GOLGA8Q	HR
996	MRPL10	BMP1
997	DENND4C	SLC39A14
998	TMED9	CDCA2
999	FANK1	DPYSL2
1000	SLC6A8	SCARA3
1001	PACC1	PBK
1002	RHBDL1	SCARA5
1003	POF1B	INTS9
1004	ZGPAT	CLU
1005	RASD1	MAK16
1006	FOXQ1	PRKDC
1007	ELOVL7	RGS20
1008	TUBG2	BPNT2
1009	BBS2	ARMC1
1010	S1PR2	VXN

1011	WBP4	NCOA2
1012	IL12A	LACTB2
1013	GOLGA6L4	TRAM1
1014	SYF2	ZC2HC1A
1015	SPART	ASPH
1016	ADAMTS12	OTUD6B
1017	STX5	CDH17
1018	TRIM35	MATN2
1019	USP30	POP1
1020	DUSP14	FBXO43
1021	PELO	UBR5
1022	BICD2	FZD6
1023	SALL4	MED30
1024	PAPLN	DSCC1
1025	TBC1D10B	DEPTOR
1026	TAF1A	RAD21
1027	SPINDOC	SNTB1
1028	HOXC13	ZHX1
1029	COL11A2	ATAD2
1030	FAM83G	ANXA13
1031	MEA1	MYC
1032	AK3	CYRIB
1033	ARL8A	PHF20L1
1034	PFDN6	CHRAC1
1035	ZNF267	GPR20
1036	ATP6V1E1	PYCR3
1037	APOBEC3H	HGH1
1038	EIF4EBP2	BOP1
1039	SLX1A	TONSL
1040	TBC1D3F	MFSD3
1041	AP1G2	RECQL4
1042	STAM	ARHGAP39
1043	CCDC82	INSL4
1044	DDX3Y	GLDC
1045	REM2	NFIB
1046	TMEM38B	MLLT3
1047	USP48	MOB3B
1048	TCEA3	KIF24
1049	LIX1L	PIGO
1050	KLC2	CA9
1051	OSBPL6	RUSC2
1052	PSORS1C2	MELK
1053	ZBTB43	CNTNAP3B
1054	CNPY3	CNTNAP3C

1055	RAB12	PCSK5
1056	SCAND1	PSAT1
1057	TNFSF14	FRMD3
1058	KXD1	RMI1
1059	WFDC5	AGTPBP1
1060	ZNF579	DAPK1
1061	MOCS2	CKS2
1062	KLF6	FAM120A
1063	STX6	PTCH1
1064	RAPGEF3	ZNF367
1065	AHCYL2	FOXE1
1066	CTC1	NR4A3
1067	CFAP74	NIPSNAP3A
1068	ABCC5	SMC2
1069	SRGAP2B	CTNNAL1
1070	ELOB	SVEP1
1071	PELI1	GNG10
1072	ATL1	POLE3
1073	APLP2	ZNF618
1074	TMEM184C	PHF19
1075	ABCG4	B3GALT9
1076	DYNLT1	C5
1077	BAIAP2	TTLL11
1078	WIPF3	DENND1A
1079	RBBP5	NEK6
1080	HIC2	RABEPK
1081	ASPHD2	PKN3
1082	PLAA	ENDOG
1083	MORF4L1	DYNC2I2
1084	EXOC1	PTPA
1085	BRAP	IER5L
1086	TRIM45	ABL1
1087	ZNF354A	PRRT1B
1088	CHMP4B	ENTPD2
1089	ZNF2	SAPCD2
1090	ATF4	PLCXD1
1091	DAB2IP	ZBED1
1092	FILIP1L	GYG2
1093	TFG	ARSL
1094	MFSD13A	CLCN4
1095	TTL	WWC3
1096	STAM2	MID1
1097	MAFG	CLTRN
1098	PPM1N	NHS

1099	MOGS	ADGRG2
1100	NRBP1	EIF1AX
1101	TBC1D3I	MBTPS2
1102	CHRM4	YY2
1103	GOLGA8H	CNKSR2
1104	POLR3GL	SYTL5
1105	CHRNA3	BCOR
1106	XPNPEP1	SUV39H1
1107	LAMP2	HSD17B10
1108	MAPKAP1	SMC1A
1109	SPAG4	SPIN4
1110	FGR	AR
1111	ZNF280A	KIF4A
1112	LSM12	ERCC6L
1113	KCNG1	TAF9B
1114	WFDC2	CENPI
1115	ANKFN1	HNRNPH2
1116	TUBAL3	PGK1
1117	GPR50	TMSB15C
1118	SS18L1	TMSB15B
1119	GOLGA8N	CLDN2
1120	HIPK4	NUP62CL
1121	GJA3	SLC25A43
1122	SLC45A3	PLS3
1123	TANK	BRCC3
1124	MAST3	VBP1
1125	MKNK2	G6PD
1126	APOBEC3D	
1127	PLIN3	
1128	CCDC117	
1129	RPL38	
1130	PRDM10	
1131	TBK1	
1132	KCNQ3	
1133	ZNF554	
1134	NIPAL3	
1135	CCDC60	
1136	KIF3A	
1137	CBARP	
1138	VNN1	
1139	MAPRE1	
1140	FAM120B	
1141	ZNF768	
1142	RASA4	

1143	PREX1	
1144	CLEC11A	
1145	PARP4	
1146	SYN2	
1147	RENBP	
1148	MRPS21	
1149	DLG2	
1150	ZNF18	
1151	AMZ2	
1152	SH3GLB1	
1153	APAF1	
1154	MAPK7	
1155	SPRY1	
1156	CXXC1	
1157	MTX1	
